# Diversity of reductive dehalogenase genes from environmental samples and enrichment cultures identified with degenerate primer PCR screens

**DOI:** 10.3389/fmicb.2013.00341

**Published:** 2013-11-19

**Authors:** Laura A. Hug, Elizabeth A. Edwards

**Affiliations:** ^1^Department of Cell and Systems Biology, University of TorontoToronto, ON, Canada; ^2^Department of Chemical Engineering and Applied Chemistry, University of TorontoToronto, ON, Canada

**Keywords:** reductive dehalogenase, enrichment culture, degenerate PCR, contaminated site, bioremediation

## Abstract

Reductive dehalogenases are the critical enzymes for anaerobic organohalide respiration, a microbial metabolic process that has been harnessed for bioremediation efforts to resolve chlorinated solvent contamination in groundwater and is implicated in the global halogen cycle. Reductive dehalogenase sequence diversity is informative for the dechlorination potential of the site or enrichment culture. A suite of degenerate PCR primers targeting a comprehensive curated set of reductive dehalogenase genes was designed and applied to 12 DNA samples extracted from contaminated and pristine sites, as well as six enrichment cultures capable of reducing chlorinated compounds to non-toxic end-products. The amplified gene products from four environmental sites and two enrichment cultures were sequenced using Illumina HiSeq, and the reductive dehalogenase complement of each sample determined. The results indicate that the diversity of the reductive dehalogenase gene family is much deeper than is currently accounted for: one-third of the translated proteins have less than 70% pairwise amino acid identity to database sequences. Approximately 60% of the sequenced reductive dehalogenase genes were broadly distributed, being identified in four or more samples, and often in previously sequenced genomes as well. In contrast, 17% of the sequenced reductive dehalogenases were unique, present in only a single sample and bearing less than 90% pairwise amino acid identity to any previously identified proteins. Many of the broadly distributed reductive dehalogenases are uncharacterized in terms of their substrate specificity, making these intriguing targets for further biochemical experimentation. Finally, comparison of samples from a contaminated site and an enrichment culture derived from the same site 8 years prior allowed examination of the effect of the enrichment process.

## Introduction

Organohalide respiring bacteria utilize chlorinated hydrocarbons as terminal electron acceptors, a respiratory process driven by the activity of a class of enzymes called reductive dehalogenases (Holliger et al., 1998b; Smidt and de Vos, 2004). This microbial activity has been leveraged for remediation of groundwater contaminated with chlorinated solvents.

Reductive dehalogenases and uncharacterized reductive dehalogenase homologous genes (*rdhA*) have been identified in a wide variety of anaerobic microorganisms, including *Sulfurospirillum* (Neumann et al., 1994), *Desulfitobacterium* (Löffler et al., 1996; Christiansen et al., 1998; Miller et al., 1998; van de Pas et al., 1999), *Dehalobacter* (Holliger et al., 1998a; Tang et al., 2012b), *Dehalococcoides* (Magnuson et al., 1998; Kube et al., 2005; Seshadri et al., 2005; McMurdie et al., 2009) and a variety of other organisms from different bacterial phyla (Thomas et al., 2008; Selesi et al., 2010; Wagner et al., 2012). Only one archaeal putative reductive dehalogenase gene has been identified, from a *Ferroglobus* species (Hafenbradl et al., 1996). *Desulfitobacteria, Dehalococcoides, Dehalogenimonas* and *Dehalobacter* spp. each encode multiple *rdhA* genes (Hölscher et al., 2004; Nonaka et al., 2006; Tang et al., 2012b), with up to 36 *rdhA* present on a single genome (McMurdie et al., 2009).

The reductive dehalogenase complement within an organism, enrichment culture, or contaminated site is of critical importance for determining the potential dechlorination activity. Despite this need, only a few reductive dehalogenases have been biochemically characterized, with confirmed activity on specific halogenated substrates [see Hug et al. (2013) for a review]. Each characterized reductive dehalogenase has spawned molecular tools for identifying and tracking closely related homologs that may share the known substrate range (Rhee et al., 2003; Krajmalnik-Brown et al., 2007; Lee et al., 2011). These approaches typically involve the use of PCR or quantitative PCR (Cupples, 2008; Bisaillon et al., 2010; Marzorati et al., 2010; Maillard et al., 2011), and allow some predictive power over what dechlorination, if any, may occur naturally at a contaminated site. This predictive ability is limited to the handful of genes with known function.

Several molecular tools have been described for examining a broader range of *rdhA* genes in a given sample (site, isolate, or culture), including both microarray-based methods and PCR-based protocols (West et al., 2008; Cheng and He, 2009; Wagner et al., 2009; Chow et al., 2010). The most commonly used primer sets are one pair (RRF2 and B1R) designed to amplify *Dehalococcoides rdhA* genes (Hölscher et al., 2004), and two sets designed to target the *pceA* and *cprA* genes from the *Desulfitobacterium* genus, respectively (Von Wintzingerode et al., 2001; Regeard et al., 2004). These primer sets have proven useful for identifying numerous *rdhA* genes from a diversity of samples (e.g., Waller et al., 2005; Futagami et al., 2009), but suffer from distinct limitations. The *Dehalococcoides*-based RRF2/B1R set's reverse primer is anchored in a downstream, associated gene, *rdhB* (Hölscher et al., 2004). It thus presupposes the *rdh* operon structure is consistently A-B, which genome sequences have shown is not always the case (McMurdie et al., 2009). The *Desulfitobacterium* primer sets distinguish two genes of known function, but do not allow more general detection of lower-similarity homologs. In both cases, the primer sets were designed based on limited reference sets available at the time, which do not adequately encompass the current known *rdhA* sequence diversity. Identification of novel, less similar reductive dehalogenases has primarily come from genome and metagenome sequencing efforts, rather than the use of clone libraries or array-based methods.

In contrast to conventional clone library or metagenomic sequencing, amplicon sequencing leveraging next-generation sequencing allows high-throughput examination of PCR products, and provides deep resolution of amplified genes of interest. Amplicon sequencing has primarily been used as a method for determining microbial diversity from environmental samples using the 16S ribosomal RNA gene and universal PCR primers (e.g., Huber et al., 2007; Fulthorpe et al., 2008). Taxonomic profiling using amplicon sequencing has been expanded to single-copy, well-conserved protein genes, including the mitochondrial Cytochrome Oxidase subunit I (Ramirez-Gonzalez et al., 2013). However, the potential of amplicon sequencing is not limited to taxonomic investigations: it represents an inexpensive, broad characterization technique for examining the diversity and distribution of functional gene families of interest.

Here we describe the design, testing, and application of a degenerate PCR primer suite for amplification of the currently known reductive dehalogenase sequence diversity. Post-amplification, we sequenced the *rdhA* gene complement of six mixed microbial samples: four from environmental sites, and two from enrichment cultures originally derived from contaminated sediment. The newly sequenced *rdhA* genes are compared to existing information for the sites/cultures, and used to examine the differences between microbial communities from environmental samples and enrichment cultures.

## Materials and methods

### Reductive dehalogenase gene tree and primer design

**Figure 1:**
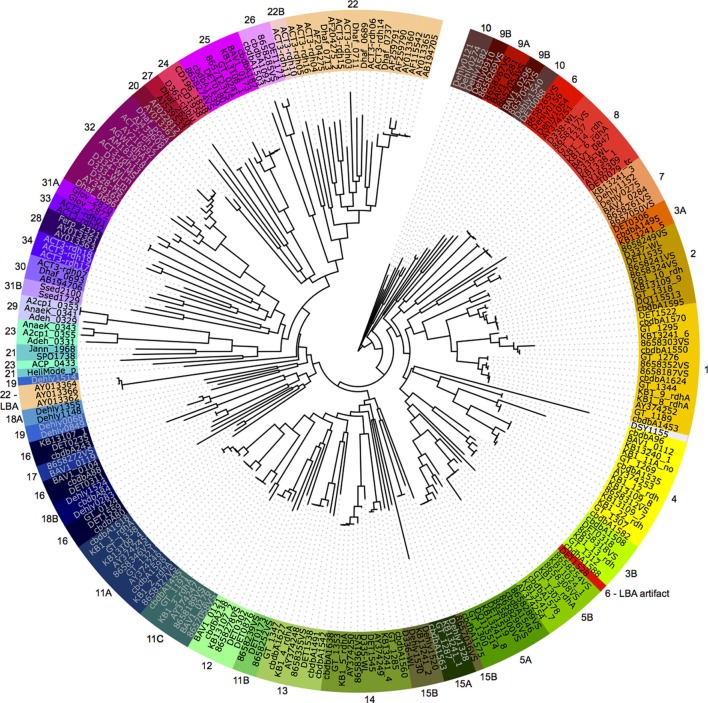
**The 44 phylogeny-derived groups for degenerate PCR primer design.** Maximum likelihood phylogeny of 255 reductive dehalogenases and reductive dehalogenase homologous genes based on a nucleotide alignment. The genes are colored by group, where each group was separately realigned and primer sets designed. Group numbers were assigned arbitrarily during the primer suite development. Two instances of long-branch attraction (LBA) within the global tree are labeled; based on smaller alignments, the sequences do belong to the assigned groups. Organism names and accession numbers are available in supplemental Table S3.

All *rdhA* genes from known organisms (where “known” required that a 16S ribosomal RNA gene sequence is available) were gathered from in-house datasets, NCBI, and the JGI IMG portal. The genes were aligned using Muscle version 3.8.31 (Edgar, 2004) and the alignment iteratively refined using Hmmer version 2.3.2 (Finn et al., 2011). The alignment was manually curated and masked in Geneious (Drummond et al., 2012). The final alignment contained 255 reductive dehalogenase genes, and comprised 4236 unambiguously aligned positions. Ten maximum likelihood trees were run from independent starting positions using RAxML HPC version 7.0.3 under the GTR+ γ model of nucleotide substitution (Stamatakis, 2006). The tree with the best likelihood was chosen for display (Figure 1).

The final reductive dehalogenase gene tree was divided into 44 sub-groups based on branch-lengths and nucleotide similarities (Figure 1). Each sub-group was realigned using Muscle version 3.8.31 (Edgar, 2004), and degenerate primers designed to target the entire sub-group using the IDT Oligoanalyzer. Primers were required to have annealing temperature ranges of 40–65°C, with hairpin melting temperatures no greater than 25°C and predicted primer dimers with a maximum of one-third of the total ΔG (See Table S1 for primer sequences, melting temperature ranges, and expected length of amplified target).

An additional six reductive dehalogenase sequences recently deposited into public databases were added to the dataset after group selection and primer design was completed. Predicted amplification by the primer groups for the six genes was tested *in silico*. In four cases, the degenerate primers contained a perfect match to the new sequence. For the remaining two genes a small number of mismatches existed. For both, the mismatches were of the nature that amplification would still be expected given the permissive conditions of the PCR reaction.

### Reductive dehalogenase gene amplification and sequencing

Primer performance was tested using pure-strain DNA from *Dehalococcoides mccartyi* VS (Müller et al., 2004; McMurdie et al., 2009), and with community DNA from the DonnaII and KB-1 consortia (Fennell et al., 1997; Duhamel and Edwards, 2006, 2007; Hug et al., 2012). All PCR reactions contained 1X *Taq* reaction buffer (NEB Inc.), 0.25 μM dNTPs, 0.5 μM each of forward and reverse primers, and 5 U of *Taq* DNA polymerase (NEB Inc.) in a total volume of 50 μL. Sample DNA amounts and concentrations are listed in Table 1. Reaction conditions for true-positive detection of all controls were determined. The following conditions were subsequently applied to all described samples: an initial denaturation at 95°C for 5 min, three cycles of denaturation at 95°C for 30 s, primer annealing at 38°C for 30 s, and elongation at 72°C for 90 s, three cycles of amplification denaturation at 95°C for 30 s, primer annealing at 45°C for 30 s, and elongation at 72°C for 90 s, denaturation at 95°C for 30 s, primer annealing at 50°C for 30 s, and elongation at 72°C for 90 s, and a final extension at 72°C for 10 min. PCR reaction conditions were purposefully permissive, where false positives were weighted less heavily than false negatives during optimization. This was to maximize the primer suite's ability to detect novel genes, for which the primers may not represent a perfect match even given degeneracies. All PCR reactions contained a single set of primers; for each DNA sample the 44 primer sets were tested concurrently during the same thermocycler run.

**Table 1:** **Samples tested with the reductive dehalogenase primer suite**.

**Sample name (substrate)**	**Initial sample type**	**DNA (ng/rxn)**	**Dechlorinating organisms (expected)**	**Amplification (# positive/44 total)**
**CONTROLS**				
KB-1 (TCE)	30 mL liquid culture	38.3	*Dhc, Geobacter*	Yes (100% TP)
DONNAII (TCE)	archived DNA extraction	15.2	*D, mccartyi* DE195	Yes (70% TP)^*^
VS (VC)	DNA extraction	20.0	*D, mccartyi* VS	Yes (100% TP)
ACT-3 (1,1,1-TCA)	30 mL liquid culture	21.4	*Dhb* (2 strains)	Yes (100% TP)
**UNKNOWNS**				
WL_culture (1,1,2-TCA)	30 mL liquid culture	10.8	*Dhb* and *Dhc*	Yes (16/44)
WL site (groundwater) (unknown)	1 L groundwater	7.8	?	Yes (13/44)
WL site (core sediment) (unknown)	5 g soil	8.9	?	No
WBC-2 1T1 (TeCA, *cis*DCE, 1,1,2-TCA)	30 mL liquid culture	7.1	*Dhb, Dhc*, and *Dehly*	Yes (20/44)
WBC-2 1T2 (1,1,2-TCA)	30 mL liquid culture	20.6	*Dhb* and *Dhc*	Yes (30/44)
WBC-2 1T3 (TeCA)	30 mL liquid culture	22.7	*Dhb, Dhc*, and *Dehly*	Yes (30/44)
WBC-2 1T4 (*cis*DCE)	30 mL liquid culture	7.8	*Dhc*	Yes (24/44)
ISSO-biostim. PMLA3_Oct09 (TCE)	DNA from SiREM	0.1 μL	*Dhc* + ?	No
ISSO-biostim. OBH19-A1Oct09 (TCE)	DNA from SiREM	3.72	*Dhc* + ?	Yes (16/44)
ISSO-biostim. OBH19-A2Oct09 (TCE)	DNA from SiREM	1.44	*Dhc* + ?	Yes (6/44)
ISSO-bioaug. PMLA3_Feb2010 (TCE)	DNA from SiREM	0.1 μL	*Dhc* + ?	Yes (16/44)
SiREM KB-1 enrichment culture (TCE)	DNA from SiREM	23	*Dhc + Geobacter*	Yes (20/44)
Toronto brickworks bulrushes (pristine)	5 g sediment	31.0	?	No
Toronto brickworks sediment (pristine)	5 g sediment	20.9	?	No
Lake Faro sediment (Messina, Italy) (pristine)	DNA from Mediterranean Group	22.5	?	Yes (15/44)
Haven tanker sediment 1 (coast off Genoa, Italy) (unknown)	DNA from Mediterranean Group	31.0	?	No
Haven tanker sediment 2 (coast off Genoa, Italy) (unknown)	DNA from Mediterranean Group	25.3	?	No
SY03 (Polluted sediments, Priolo Italy) (unknown)	DNA from Mediterranean Group	21.6	?	No

Sample type and DNA amount (where available) are listed as well as the expected dechlorinating organisms within the sample and the amplification results. TP, true positive, Dhc, Dehalococcoides sp., Dehly, Dehalogenimonas sp., Dhb, Dehalobacter sp.

* Genomic DNA from D. mccartyi 195 was heavily degraded, and a best-case amplification of 70% of expected rdhA was accepted.

A total of 23 samples were tested with the reductive dehalogenase primer suite (Table 1). DNA from strain VS (Stanford), the DonnaII consortium (Cornell), a site undergoing biostimulation and bioaugmentation in Southern Ontario (ISSO, SiREM), and Mediterranean sediments (Italy) were generously provided by Dr. Alfred Spormann (Stanford), Dr. Ruth Richardson (Cornell), SiREM labs, and Violetta La Cono (IAMC, Messina, Italy). DNA samples from the KB-1, WL, WBC-2, and TCA/MEL consortia, as well as the WL and brickworks sites were extracted in house using the MoBio PowerClean soil extraction kit following the alternative protocol for maximum yields. Sample amounts used in DNA extractions are listed in Table 1.

### Cloning

Select PCR reactions from the KB-1 consortium were cleaned using the Fermentas PCR spin-column clean-up kit with the cleaned product eluted in 35 μL of ddH_2_O instead of elution buffer. PCR products were cloned into the TOPO-TA vector (Sigma) and transformed into TOP-10 chemically competent *E. coli* (Sigma) under manufacturers directions. Colonies were grown on LB agar plates containing 50 mg/mL kanamycin and 40 mg/mL X-gal. White colonies were screened through PCR with the vector primers T7F and M13R (Sigma). For each reaction, five representative colony PCR reactions with the correct size of insert were sent for Sanger sequencing in both forward and reverse directions with the vector primers (service provided by the The Center for Applied Genomics, The Hospital for Sick Children, Toronto, Canada, Toronto). Sequences were assembled into contigs where possible, and identified using BLAST.

### Illumina sequencing, assembly, and analysis

**Figure 2:**
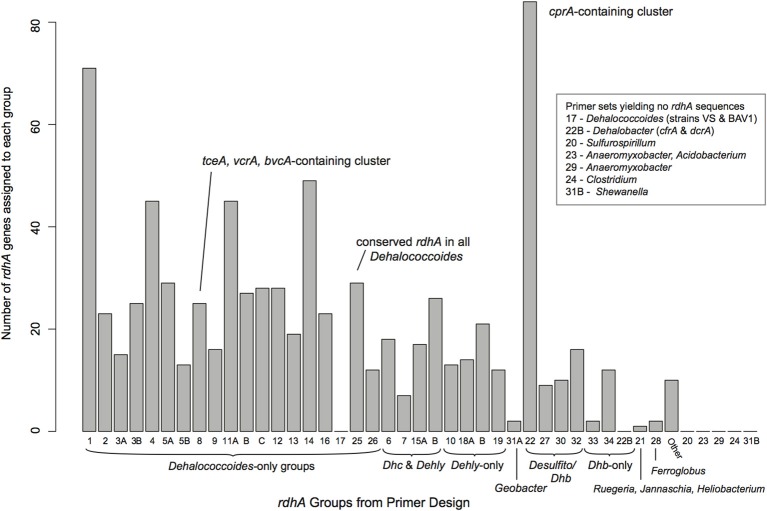
**Distribution of identified *rdhA* genes from the sequenced samples in relation to the RDase primer groups.** *rdhA* genes identified were primarily related to *Dehalococcoides, Dehalogenimonas, Dehalobacter*, and *Desulfitobacterium rdhA* genes. Group numbers are arbitrary based on the process of primer design. See Table S1 for the suite of primers used and their sequences.

Six samples showing large numbers of positive PCR reactions were chosen for Illumina sequencing. All reactions showing amplification were cleaned using the Fermentas PCR spin-column clean-up kit with five reactions pooled into each clean-up spin filter, and the final cleaned products eluted with 50 μL of elution buffer. All cleaned PCR products from a single DNA sample were pooled together, and barcoded for Illumina sequencing using the TruSeq DNA Sample Preparation Kit (Illumina) under the manufacturer's recommended protocol for a library with 200–300 bp inserts from genomic DNA, excluding optional gel purification steps. The six barcoded sequencing libraries were pooled and sequenced with a single lane of Illumina HiSeq sequencing (service provided by the The Center for Applied Genomics, The Hospital for Sick Children, Toronto, Canada, Toronto). The sequences were deconvoluted by barcodes, and pre-processed using tools available on the Galaxy-JGI server. All reads were filtered using Artifact Trimmer to remove low quality, adapter-containing, and low-complexity reads. Passing reads were trimmed to a length of 77 base pairs (4 bases from 5′ and 21 base pairs from 3′ end), criteria based on overall quality statistics. Assemblies were conducted using idba_ud under default parameters (Peng et al., 2012). All contigs were searched against nucleotide and translated curated reductive dehalogenase datasets using BLASTn and BLASTx, and contigs longer than 500 base pairs were searched against the NCBI nr database using BLASTx with a minimum *e*-value of 1e^−10^. The identified *rdhA* genes were assigned to a primer group based on their best blast-match from the curated *rdhA* database (Figure 2). The putative *rdhA* gene sequences were screened for chimeras using blastn and blastx searched against the NCBI databases. All *rdhA* genes were translated, and protein sequences longer than 200 aa were included in phylogenetic trees for placement with the curated RdhA dataset (see supplemental Figure S1 for all trees). All sequences sharing ≥90% pairwise amino acid identity (PID) with proteins from the defined reductive dehalogenase ortholog groups [RD_OG, (Hug et al., 2013)] were noted (Table S2). As the host organisms for the *rdhA* genes were not identified, the sequences have not been incorporated into the formal classification system, which is used here solely to anchor the novel sequences within the current known diversity.

All reductive dehalogenase sequences >200 nucleotides long have been deposited in the NCBI nr database under accession numbers KF138603–KF139342 (Table S2).

## Results

### *rdhA* primer suite

A universal primer suite for detection of *rdhA* genes was developed based on a curated reductive dehalogenase dataset containing 261 sequences (Hug et al., 2013). The primer suite is composed of 44 sets of degenerate primers targeting phylogenetically-related groups of *rdhA*s (Figure 1). It was designed such that all primer sets amplify under common conditions, and can be tested simultaneously on DNA samples of interest. The primer suite was optimized using 4 different *Dehalococcoides mccartyi* strains, one *Geobacter* strain, and two *Dehalobacter* strains, which was the diversity of genomically-characterized dechlorinating organisms available. Reaction conditions and primer sequences were tested and redesigned iteratively until 100% true positive detection for the controls was accomplished.

For one of the positive control DNA samples, the KB-1 mixed microbial consortium, existing clone libraries and metagenome sequencing may not have identified all *rdhA* genes present (Waller et al., 2005; Hug et al., 2012). The final reaction conditions resulted in amplification in all expected PCR reactions (11 reactions) for the KB-1 consortium sample. However, an additional 18 unexpected positive reactions were observed. These 18 reactions were cloned and sequenced. Five of the cloned PCR reactions presented clean, sharp bands on an agarose gel, and were identified as follows: a previously unidentified *rdhA* gene homologous to an *rdhA* from *Dehalococcoides mccartyi* CBDB1 (cbdbA1092, 100% pairwise identity), two non-group-specific amplifications of known *rdhA* genes from the KB-1 culture, one iron-sulfur cluster domain-containing protein from a *Dehalococcoides* (homology to DhcVS_1285, accession YP_003330713), and a reaction containing phosphatase and methyltransferase sequences from *Dehalococcoides*. The remaining 13 reactions were visualized as ladders of bands or smears of amplified DNA on an agarose gel. Two of these reactions failed to yield sufficient DNA for cloning. In the remaining eleven reactions, clone sequencing determined that the products were due to non-specific amplification, and included phosphatases, methylases, oxidoreductases, helicases, a metallobetalactamase, and hypothetical proteins, from *Dehalococcoides, Geobacter*, and other, non-dechlorinating organisms in the KB-1 consortium. From this, the performance of the primer suite was deemed satisfactory: it had detected all of the known *rdhA* genes within positive controls, and, for the KB-1 consortium, it allowed identification of one previously unknown *rdhA* gene. The presence of non-specific amplification was expected given the degenerate nature of the primer sequences. The primer suite was expressly designed to be permissive, in order to identify divergent *rdhA* genes.

The primer suite was subsequently used to examine 18 uncharacterized samples including DNA from active bioremediation field sites, pristine environmental sites, and in-house enrichment cultures capable of reductive dechlorination of a variety of halogenated compounds (Table 1). Six samples of interest were chosen for sequencing based on high numbers of positive amplification reactions. Two paired samples sent for sequencing were groundwater from a chlorinated solvent-contaminated field site in West Louisiana (“WL site”) and an enrichment culture derived from the same site 8 years prior, which had been enriched for dechlorination of chlorinated ethanes (“WL culture”) (Grostern and Edwards, 2006). A second pair of samples came from a contaminated industrial site in Southern Ontario (ISSO), where trichloroethene (TCE) was historically used as a degreasing agent. The ISSO site is characterized by high levels of chlorinated ethene contamination (TCE and daughter products) along with fractured bedrock aquifers that allow high rates of groundwater flow. The site was biostimulated in 2008 with amendment of electron donor to stimulate the activity of natively occurring dechlorinating organisms, and later bioaugmented in 2009 with the KB-1 consortium. The site was screened with the primer suite across several stages of the bioremediation effort. Cleaned PCR reactions from two post-biostimulation groundwater samples, from different depths of the same well taken in October 2009, were pooled and sent for sequencing (“ISSO-biostim”), along with one post-bioaugmentation groundwater sample taken in February 2010 (“ISSO-bioaug”). Four dechlorinating enrichment cultures derived from a contaminated site in Maryland were screened with the primer suite (WBC-2 1T1 through 1T4, (Manchester et al., 2012). The WBC-2 1T3P subculture chosen for sequencing has been maintained with 1,1,2,2-tetrachloroethane (TeCA) as the sole electron acceptor for over 5 years, creating a strong enrichment of three dechlorinating organisms: a *Dehalobacter* sp., a *Dehalococcoides mccartyi* strain, and a *Dehalogenimonas* sp. (“WBC-2”) (Manchester et al., 2012). The final sample sent for sequencing was sediment from the putatively pristine brackish Lake Faro near Messina, Italy (“Lake Faro”).

### Illumina sequencing and *rdhA* gene identification

All samples were sequenced on a single lane of Illumina paired-end sequencing. The sequencing generated 4.54 million 77 bp reads for a total of 350.2 Mbp post-quality trimming and processing, distributed approximately evenly across the six barcoded samples (average 58 Mbp per sample, standard deviation 8.2 Mbp). As expected, putative reductive dehalogenases were present in the samples' assemblies, as were non-specific sequences. The sample assemblies contained 2545–24,981 contigs above a 250 bp length cut-off, and included between 68 and 208 *rdhA* genes (Table 2). A total of 798 *rdhA* sequences were identified from the six samples, 349 of which represent partial predicted proteins with a minimum length of 200 aa. Thirty-seven of the 44 primer groups are represented within the 798 *rdhA* genes, with the majority of genes identified being most closely related to *Dehalococcoides, Dehalogenimonas, Dehalobacter*, and *Desulfitobacterium rdhA* genes (Figure 2). Two *Geobacter*-type *rdhA* genes were identified from the “ISSO-biostim” sample. Several of the primer groups that are not represented contain predicted *rdhA* genes from known dechlorinators, including *Sulfurospirillum, Anaeromyxobacter*, and *Shewanella* sp.

**Table 2:** **Summary statistics for sequence assemblies and distribution of homologs to substrate-characterized reductive dehalogenases in each sample**.

	**Organism**	**Substrates**	**ISSO-biostim**	**ISSO-bioaug**	**WBC-2**	**WL site**	**WL culture**	**Lake Faro**
# primer rxns			16 and 6 (pooled)	16	30	13	16	15
bp sequenced			60,405,642	59,819,232	55,802,048	60,920,614	44,101,316	69,219,680
Contigs >250 bp			24,981	16,141	2924	14,428	2545	11,200
# *rdhA* (>200 aa)			208 (73)	144 (53)	140 (69)	156 (69)	82 (42)	68 (43)
*vcrA* Müller et al., 2004	*D. mccartyi* VS	VC, *cis*-DCE, TCE	KF138765 (335, 96.4%)	KF138616 (391, 96.2%)	KF138993 (466, 97.2%)		KF139115 (466, 98.1%)	
*bvcA* Krajmalnik-Brown et al., 2004	*D. mccartyi* BAV1	VC, *cis*-DCE, 1,2-DCA	KF138745 (393, 97.5%)	KF138603 (500, 97.6%)	KF138998 (452, 97.8%)			KF138942 (289, 97.6%)
*tceA* Magnuson et al., 2000	*D. mccartyi* 195	TCE	KF138766 (330, 94.5%)	KF138615 (399, 95.5%)	KF138991 (497, 96.2%)	KF139194 (480, 96.0%)	Partial	KF138917 (501, 96.0%)
*pceA* Magnuson et al., 1998	*D. mccartyi* 195	PCE		KF138713 (80, 93.8%) KF138714 (80, 95%)	KF139013 (399, 92.7%)	KF139222 (361, 92.5%)	KF139135 (332, 91.3%)	KF138924 (350, 92.3%)
*cbrA* Adrian et al., 2007	*D. mccartyi* CBDB1	TeCB, TCB, PeCB		KF138672 (146, 98.6%)				
*mbrA* Chow et al., 2010	*D. mccartyi* MB	TCE	KF138786 (276, 98.9%)	KF138648 (238, 99.2%)	KF139042 (280, 98.9%)	KF139289 (123)	KF139150 (226, 92.5%)	KF138927 (345, 99.1%)
*dcrA* Tang and Edwards, 2013	*Dehalobacter* sp. strain DCA	1,1-DCA			KF139036 (317, 93.4%)			
*dcrA* Grostern and Edwards, 2009	*Dehalobacter* sp. strain WL	1,2-DCA		KF138717 (79, 97.5%)		KF139300 (110, 97.3%)	KF139177 (100, 97.0%) KF139137 (315, 98.4%)	
*pceA* Wagner et al., 2012	*G. lovleyi* sp.	PCE	KF138753 (363, 97.0%)					
*pceA* Maillard et al., 2003	*D. restrictus* PER-K23 *D. hafniense* TCE1	PCE	KF138741 (405, 96.5%)	KF138650 (236, 94.1%)	KF139003 (436, 95.6%)	KF139245 (252, 95.6%)		KF138921 (392, 96.4%)

For each homolog, the sequence length (amino acids) and percent identity to the characterized enzyme is noted in brackets.

VC, vinyl chloride, DCE, dichloroethene, TCE, trichloroethene, PCE, tetrachloroethene, DCA, dichloroethane, TeCB, 1,2,3,4-tetrachlorobenzene, TCB, 1,2,3-trichlorobenzene, PeCB, pentachlorobenzene.

The number of non-specifically-amplified sequences was likely due to two factors: first, all positive PCR reactions were sequenced, even if they presented smeared or laddered bands rather than clear defined bands on an agarose gel, and second, the depth of sequencing conducted on these samples was such that sequences present at very low levels within the cleaned PCR products were expected to be completely sequenced. The depths of coverage for the assembled datasets reveal significant enrichment of *rdhA* gene sequences. The average coverage for non-*rdhA* contigs ranged from 54 to 291x for five of the six samples, in contrast to an average coverage for *rdhA* contigs of 1868–11,011x. The only sample for which *rdhA* contigs were not specifically enriched was Lake Faro, the only putatively pristine environment sequenced (average coverage of 37x for *rdhA* contigs compared to 157x for all contigs). This indicates *rdhA* genes may be at much lower abundance in this sample, and thus both amplification and deep sequencing were required for identification of *rdhA* from this pristine environment, in the absence of any enrichment for dehalogenating activity.

### Homologs to characterized reductive dehalogenases

**Figure 3:**
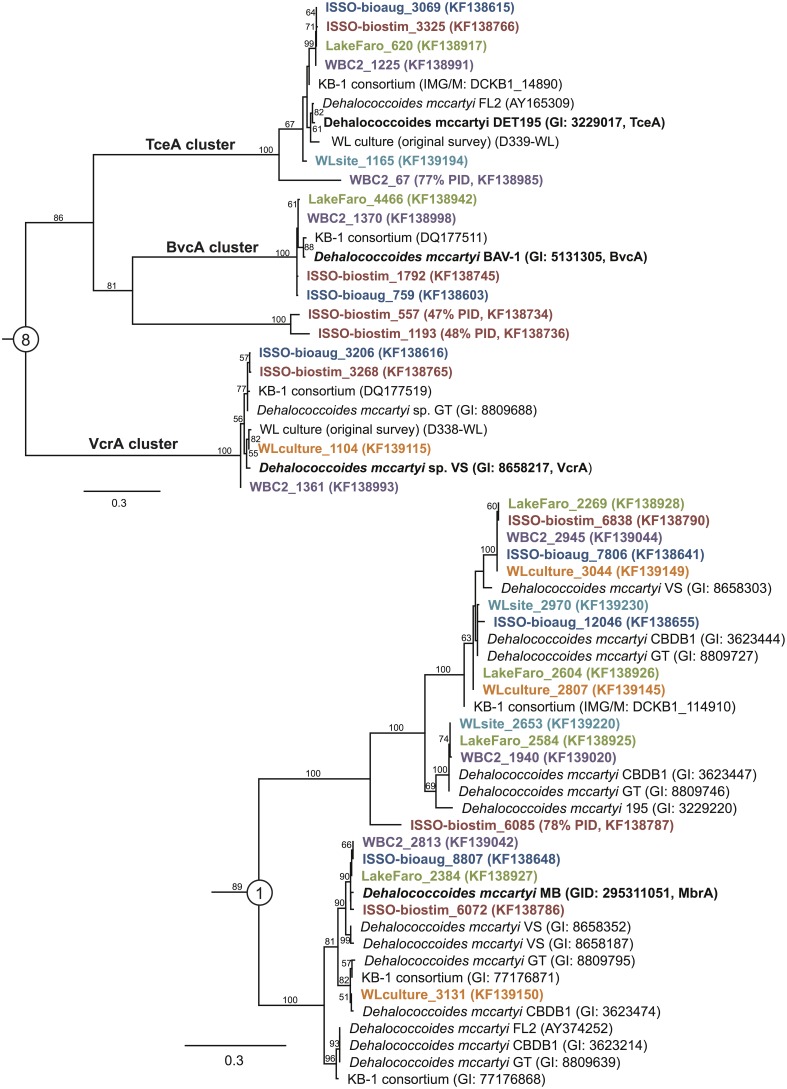
**Reductive dehalogenase homologs are broadly distributed, including homologs to enzymes of known function.** Maximum likelihood trees of newly sequenced RdhA with best homology match to RdhA sequences from primer groups 8 and 1, including the characterized chloroethene dehalogenases BvcA, VcrA, and TceA (group 8) and MbrA (group 1). The trees were generated in Phyml (Guindon and Gascuel, 2003) under the LG + γ model of amino acid evolution, from a Muscle v. 3.8.31 alignment containing an outgroup sequence (not shown) (Edgar, 2004). Names for newly sequenced RdhA genes correspond to the contig from the idba_ud assemblies (Peng et al., 2012), with NCBI accession numbers in parentheses.

The newly-sequenced *rdhA* genes were translated and aligned with their closest known homologs and the percent amino acid identity between known and newly-sequenced RdhA proteins calculated. All six of the sequenced samples contain predicted reductive dehalogenases with >90%, and in many cases, >97% sequence identity to characterized reductive dehalogenases whose substrate specificities are known (Table 2). All six samples contain close homologs to the *D. mccartyi*-associated trichloroethene-degrading enzymes TceA and MbrA (Magnuson et al., 2000; Chow et al., 2010) (Figure 3, Table 2). All six samples also contain a homolog to a characterized tetrachloroethene-dechlorinating enzyme, though the taxonomic affiliations of the genes differ. The ISSO-biostim sample contains the sole homolog to the *Geobacter* PceA RDase, while the remaining five samples contain a homolog to the *D. mccartyi* PceA (Magnuson et al., 1998; Tang et al., 2012a). Additionally, a Firmicute-associated PceA homolog is present in all but the WL culture sample. Dichloroethane dehalogenases (DcrAs) are present in the ISSO-bioaug, WBC-2, and both WL samples, while the ISSO-bioaug sample contains the only homolog to the chloroaromatic-dechlorinating CbrA from *D. mccartyi* CBDB1 (Adrian et al., 2007).

The WL site is the only sample lacking a homolog to a vinyl chloride (VC) reductase, while the ISSO samples and the WBC-2 sample contain homologs to both known VC reductases, BvcA and VcrA (Krajmalnik-Brown et al., 2004; Müller et al., 2004) (Figure 3). One of the notable aspects of the bioremediation effort at the ISSO site is that dechlorination was stimulated by electron donor amendment prior to bioaugmentation with the commercial enrichment culture KB-1®. The presence of VcrA and BvcA homologs in the pre-bioaugmentation sample provides a genetic mechanism for the VC dechlorination activity observed in the biostimulated bedrock. Bioaugmentation with KB-1 increased rates of dechlorination, which may have been due to an increased dosage of the pertinent functional genes, the activities of the supporting microbial community in the KB-1 culture (Duhamel and Edwards, 2007; Hug et al., 2012), introduction of an as-yet uncharacterized but functionally important RdhA, or a combination of the three.

Notably absent from all six samples were any homologs with >90% ID to Firmicute enzymes active on chlorinated aromatic compounds (CprA, PrdA) or the PceA from *Sulfurospirillum* sp. Within the known, characterized *rdhA* genes, sequence identity above 90% can accompany a significant shift in substrate specificity [i.e., *cfrA* and *dcrA* from *Dehalobacter* sp. strains CF and DCA Tang and Edwards, 2013], meaning assigned substrate specificities, even at this highly conserved level of identity, are putative and require experimental confirmation.

### Distribution of novel and uncharacterized *rdhAs*

**Figure 4:**
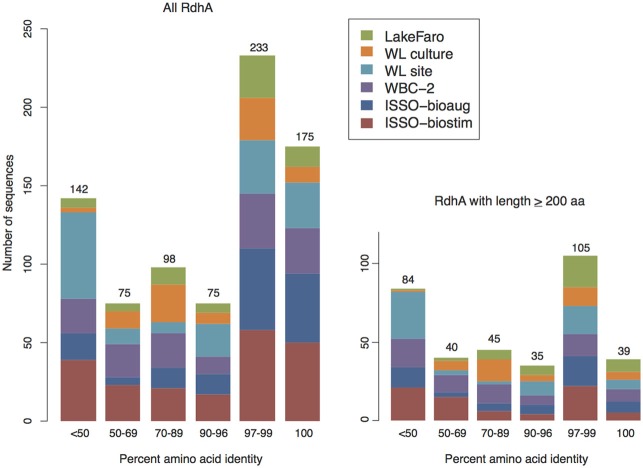
**Newly sequenced RdhA proteins are split between close homologs to known sequences and novel sequences.** Barcharts of percent amino acid identity between the newly-sequenced RdhAs and the most closely related RdhA from available protein databases (translated in-house curated *rdhA* database as well as NCBI Protein non-redundant database). Protein ID was based on perfect amino acid matches for best-match alignments spanning greater than 90% of the RdhAs from this study. Left: all RdhAs derived from this study, right: RdhAs longer than 200 aa (included in phylogenetic analyses). See Table S2 for comparison results of all new RdhA sequences against the curated RdhA and NCBI nr databases.

**Figure 5:**
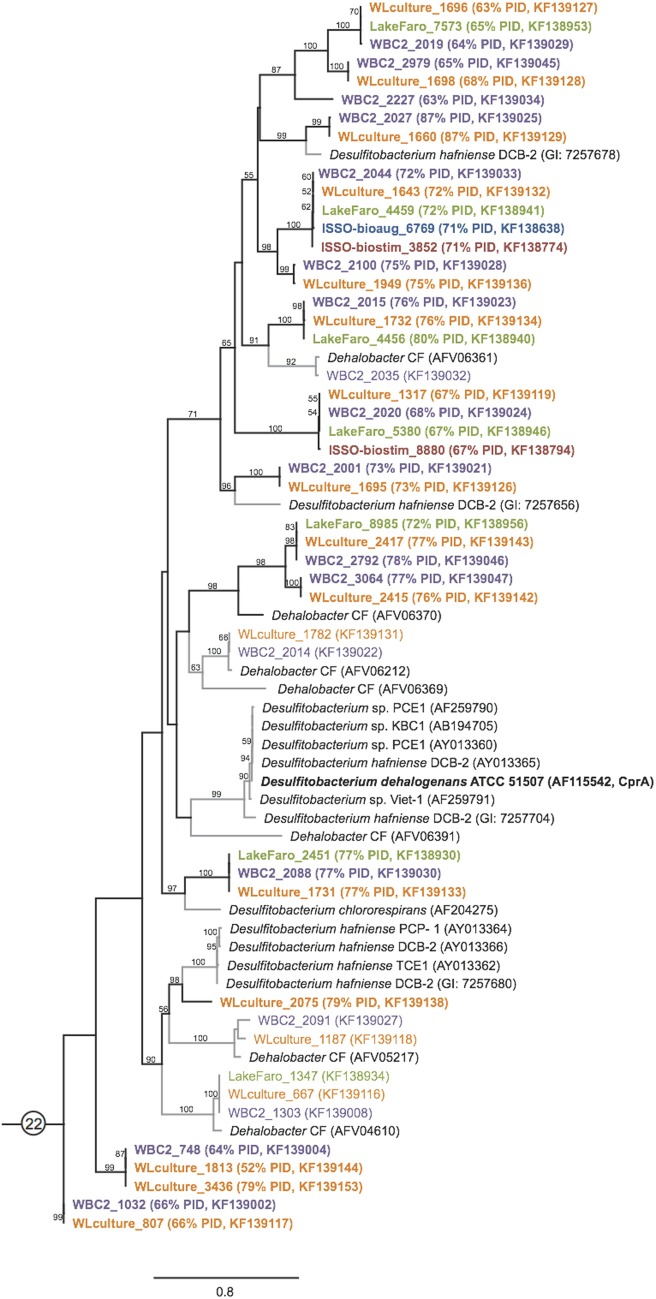
**PCR primer suite derived sequences expand the known diversity of reductive dehalogenase homologous genes.** Maximum likelihood tree of RdhA sequences from primer group 22, including the characterized chloroaromatic dehalogenase CprA. The tree was generated as described in Figure 3. Sequences from the WBC-2 and WL mixed microbial consortia and the pristine sediment from Lake Faro indicate presence of numerous previously unknown reductive dehalogenase clades with less than 80% sequence identity to previously sequenced Firmicute RdhAs. Novel RdhA are highlighted with bold type and black branches on the tree.

The vast majority of the 798 newly sequenced *rdhA* genes either bear significant sequence similarity to uncharacterized *rdhA* genes with no known substrate specificity, or represent divergent sequences with no closely related database sequence. Approximately one quarter of the *rdhA* genes identified are perfectly identical on an amino acid level with known RdhA (175 sequences), while one third share 90–99% identity with their closest known homolog (310 sequences) (Figure 4). The remaining ~40% of the RdhA sequences are more novel sequences, sharing only 30–89% amino acid identity with any known RdhA (313 sequences, 241 new RdhA types based on clustering at 90% ID). The different levels of sequence similarity can be observed within the phylogenies of sequences affiliated with primer groups 8 and 1, both groups containing *Dehalococcoides*-derived chlorinated ethene-active RDases (Figure 3). The identified sequences that are highly similar to the RDases of known function were discussed above. Notable on the primer group 8 phylogeny are three sequences with low sequence identity to the characterized enzymes: one RdhA sequence from the WBC-2 enrichment culture is related to the TceA cluster with only 77% PID, and two RdhA from the “ISSO-biostim” place as a separate clade branching off of the BvcA cluster, sharing only 47–48% PID with BvcA from *Dehalococcoides mccartyi* BAV-1. All three sequences are near-complete (579, 456, and 430 aa), meaning this is true sequence divergence rather than a long branch artifact from a partial sequence. In all three cases, the low sequence identity precludes a prediction of substrate specificities, but this clade of sequences is specifically active on chlorinated ethenes, suggesting both a putative substrate range and that these genes may be important future targets for classification. Within the primer group 1 phylogeny are sequences homologous to the MbrA TCE-dechlorinating enzyme, as well as a large clade of known and newly determined sequences with no known substrate specificity. The uncharacterized reductive dehalogenase group shares >90% PID within all members, and contains sequences from isolate strains (*D. mccartyi* CBDB1, 195, VS, and GT) as well as from all six of the samples from this study. This clade represents broadly distributed reductive dehalogenases, implying the proteins are likely functionally important to the organisms encoding them. The RdhA sequences affiliated with primer group 22, containing the chlorophenol-active CprA from *Desulfitobacterium dehalogenans*, present another aspect of the identified reductive dehalogenase diversity (Figure 5). This primer group amplified numerous divergent sequences, largely from the WL and WBC-2 cultures and the Lake Faro sample. Most of the new RdhA sequences from primer group 22 bear less than 80% PID to previously sequenced proteins, but share >90% PID with other sequences from this study. These sequences represent broadly distributed, previously unknown reductive dehalogenases. The wide taxonomic diversity of closely-related CprA homologs from the NCBI database can be attributed to the existence of CprA-specific primers for amplification of the known functional gene (Von Wintzingerode et al., 2001). In contrast, the identified RdhAs from the primer group 22 primers indicates substantial untapped sequence diversity surrounding this characterized reductive dehalogenase.

## Discussion

The reductive dehalogenase gene family is important within the global chlorine cycle as well as being of industrial significance for remediation efforts (Krzmarzick et al., 2012). The gene family is widespread across bacterial phyla, and exhibits significant diversity across gene sequences. Most studies focus on or search for the limited number of characterized RDases within a system. While this is useful for determining the potential for known activities, these approaches will not lead to the identification of novel, potentially important, *rdhA* genes. Each newly published genome sequence has provided novel *rdhA* sequences, indicating the diversity of the gene family is nowhere near exhaustively covered.

### *rdhA* primer suite

We designed and tested a reductive dehalogenase degenerate primer suite based on a curated database of *rdhA* sequences. The *rdhA* primer suite, comprising 44 primer pairs targeting 261 *rdhA* genes, proved capable of amplifying a wide diversity of *rdhA* genes from mixed microbial samples of varying complexity. The degenerate primers and permissive PCR conditions allowed detection of *rdhA* genes with low sequence similarity to genes from the reference set, providing a broad screen of the reductive dehalogenase complement of a sample. As all 44 reactions can be conducted during a single thermocycler program, the primer suite represents a relatively low-cost, efficient method for screening a sample for the presence of *rdhA* genes and providing an initial fingerprint of the *rdhA* diversity therein. Sample bar-coding and next-generation sequencing yielded excellent coverage of the amplified products, and avoided the heavy workload and high costs associated with clone library sequencing. Illumina sequencing provided high quality reads that facilitated assembly of the amplicons, and any false-positive non-*rdhA* sequences were easily screened out at the sequence annotation step.

Moving forward with the *rdhA* primer suite, there are several improvements that can be considered. To identify genes with low sequence identity to the reference set, the amplification conditions must be permissive, and some non-specific amplification is expected and accepted. However, the low percent of *rdhA* contigs in the data suggest a more stringent approach would still yield sufficiently permissive conditions. We suggest moving forward with the primer suite using more stringent PCR conditions, stricter selection criteria when amplification products are selected for sequencing, and/or lower sequencing depth conducted. In this study, clear bands on a gel were associated with *rdhA* gene products, while smears were more likely to consist of non-specific gene products. The use of barcoded Illumina libraries allowed parallelization of sequencing samples of interest. The extremely high coverage values for reductive dehalogenase genes, and the presence of many non-*rdhA* contigs at lower coverage indicates this parallelization can be leveraged further. In future assays, a larger number of samples can be included on a shared Illumina lane, and, with better tuning of sequence depth to *rdhA* gene number, the amount of low-coverage non-specific sequence that is generated can likely be greatly reduced. In future trials with the primer suite, multiplexing the PCR reactions would significantly streamline the workflow per sample, though the advantage of conducting fewer PCR reactions must be balanced with the potential for chimeric amplification products. Finally, the primer sets may also be used independently as group-specific primer sets for targeted studies examining a specific group within the RdhA diversity.

### Reductive dehalogenase distribution and diversity

**Figure 6:**
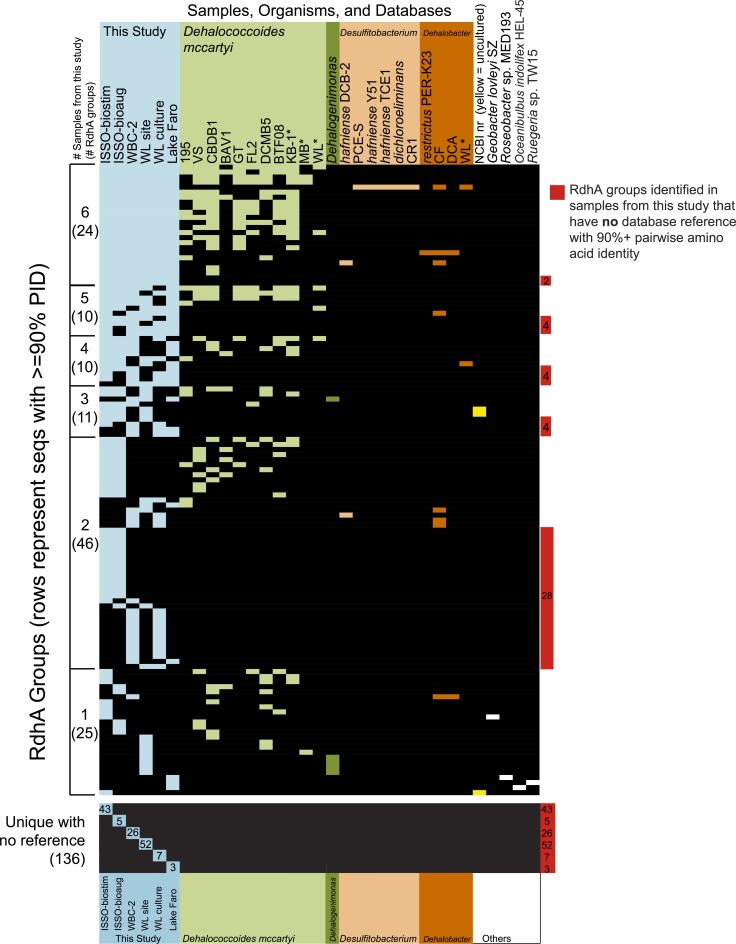
**Reductive dehalogenase homologous genes identified from the six samples are broadly distributed and highly diverse.** Presence/absence table of the distribution of all RdhA groups, as defined by 90+ % PID between all group members. The table is ordered by the number of samples from this study represented in the group, then by the total number of organisms/samples containing a group member. The first column tallies in parentheses the RdhA groups identified in 6, 5, 4, 3, 2, and only 1 of the samples analyzed in this study, while the columns provide a comparison of the groups' abundances in sequenced genomes, characterized consortia, and representation in the NCBI nr database. Samples from this study are in blue, isolate genomes and characterized mixed microbial consortia (e.g., WL and KB-1) included in the original primer suite reference set are colored by phylum, and reference genomes not included in the reference set are in white. Groups containing only sequences identified in this study are highlighted in red on the right. Cases where the sole homolog to a new RdhA sequence is an NCBI database sequence from an uncultured bacterium are marked in yellow within the NCBI nr column. Asterisks note enrichment cultures for which RdhAs have been identified, but for which the full complement of RdhAs may not be fully determined at this time. Accession numbers and further group information is available in supplemental Table S4.

Sequencing of six samples from this trial resulted in the identification of 798 putative *rdhA* genes. While homologs to several characterized reductive dehalogenases of known function were identified from each sample, the majority of sequenced genes were either related to previously sequenced uncharacterized genes, or represented novel sequences with no close homologs in existing databases. Two-thirds of the RdhA sequences from this study shared >90% PID with a database sequence. A surprising observation was that many of the *rdhA* amplified by the primer groups are broadly distributed within the environments and samples examined (Figure 6, Figure S1). The vast majority of sequences with database homologs also share >90% PID with sequences from other samples in this study. Approximately 15% of the novel RdhA sequences had close homologs within other samples from this study in the absence of related database sequences. This 15% includes 14 RdhA groups containing sequences from three or more of the six samples (60 RdhA total, Figure 6), representing broadly distributed, previously unknown RdhAs. The distribution of these RdhAs is suggestive of a ubiquitous role for these enzymes in subsurface environments, perhaps independent of anthropogenic activities. Also notable was the diversity of unique RdhAs, representing 17% of the total RdhAs identified for which no known or newly sequenced homologs exist. The two contaminated site samples, WL site and ISSO-biostim, had the highest number of unique RdhAs (Figure 6). This reflects the RdhA diversity present in native subsurface microbial communities in the presence of halogenated solvents, which is inevitably reduced during the laboratory enrichment process. In total, the six samples analyzed resulted in 241 new RdhA types: a protein or group of proteins with <90% pairwise ID to current database sequences, thus significantly expanding the current known diversity of this gene family. Two newly sequenced *Dehalococcoides mccartyi* strain's genomes [BTFO8 and DCMB5 (Pöritz et al., 2013)] allowed a comparison of the distribution and novelty of recently identified *rdhA* genes. In keeping with the results observed here, the two new strains' genomes contain some broadly distributed *rdhA* genes, including new members of 13 of the 23 groups with representatives from all six samples from this study (Figure 6). The new genomes also contain several less common RdhAs, including two from strain BTFO8 and three from strain DCMB5 whose only available homologs with >90% identity come from this study (Figure 6). *D. mccartyi* strain DCMB5 additionally contains three predicted reductive dehalogenases with <90% identity to any existing sequences, including this study. These three novel sequences include one with only 36% identity to the closest RdhA, and one whose closest homolog is an RdhA from the WBC-2 culture in this study, though with only 80% sequence identity. The identification of numerous divergent sequences within the reductive dehalogenase gene family confirms the diversity of the gene family is not nearly fully described. It remains to be seen whether the more-divergent *rdhA* genes have organohalide respiration functions, which can only be determined through activity-based experimentation. The broad distribution of many uncharacterized RdhAs suggests they are functionally important, and are interesting targets for biochemical characterization. The RdhA protein sequences elucidated here will be valuable for native PAGE proteomic analyses, which require reference sequences for peptide mapping and which have proven successful in identifying active reductive dehalogenases on specific substrates (Tang et al., 2012a).

### WL: from contaminated site to enrichment culture

The WL culture assayed here was derived from a soil microcosm sampled from a contaminated site in West Louisiana, and has been enriched with 1,1,2-trichloroethane as the sole electron acceptor for 8 years in sediment-free media. Clone libraries, quantitative PCR, and culturing work identified a *Dehalobacter* sp. and a *Dehalococcoides mccartyi* strain that work cooperatively to fully dechlorinate 1,1,2-TCA *via* VC to ethene (Grostern and Edwards, 2006). The sequences of eight *rdhA* genes (from *Dehalococcoides* and *Dehalobacter*) have been identified from the enrichment culture, using degenerate primer-based PCR amplification and sequencing (Grostern and Edwards, 2009). Here, we were able to contrast the *rdhA* genes identified from this enrichment culture with *rdhA* genes from groundwater taken in May 2011 from the same contaminated site as the original microcosm.

The WL culture RdhA bear highest sequence similarity with *Dehalobacter* sp., *Dehalococcoides mccartyi* strains, and *Desulfitobacterium* RdhA (20, 40, and 20 sequences, respectively). The last two RdhA are most similar to *D. lykanthroporepellens* sequences. Notably, none of the *Desulfitobacterium*-like and *Dehalogenimonas*-like sequences bear greater than 90% PID to reference sequences, implying these sequences may well be divergent *Dehalobacter* and *Dehalococcoides* sequences for which no closely related database sequence exists, which would fit the profile of identified organohalide respiring organisms identified in the culture. Seven of the eight known WL *rdhA* genes were identified in the WL culture sequences, all but one *Dehalococcoides mccartyi* gene.

The predicted community composition of the WL site based on RdhA affiliations is substantially different from the WL enrichment culture. The groundwater RdhA are dominated by homologs to the *Dehalococcoidia*: 105 *Dehalococcoides*-like sequences and 42 *Dehalogenimonas*-like sequences. There are 8 sequences with best matches to *Dehalobacter* sequences, 5 with *Desulfitobacterium*, 3 with *Ruegeria* sp., 1 with Deltaproteobacterium NaphS2, and 1 to the sole Archaeal RdhA, from *Ferroglobus placidus* DSM 10642. Of the lower abundance organisms, only one *Desulfitobacterium*-like RdhA has >90% PID to database sequences: all other *Desulfitobacterium, Ruegeria, Ferroglobus*, and Deltaproteobacterium NaphS2-associated sequences have low PID (<60 PID). Six of the eight previously identified *rdhA* from the WL culture were present in the WL site groundwater sample, including the one *rdhA* gene not identified in the current WL culture sample.

The absence of VcrA and the diversity of RdhA host organisms in the WL site sample distinguish it from the WL enrichment culture. These differences are likely the result of selective forces on the enrichment culture for the *Dehalobacter* sp. dihaloelimination activity on 1,1,2-TCA and *Dehalococcoides*-mediated VC reduction over the course of 8 years, with a concurrent reduction in community complexity and enrichment over time and through transfers. Despite 8 years of temporal separation and enrichment, the two sequence datasets share 39 RdhA with 99–100% PID, including many of the full-length sequences (Figure 6, Figure S1). The shared profile indicates microbial communities, or at least specific dechlorination functions, can be stable over relatively long time scales. These RdhAs have persisted within the aquifer groundwater of the WL site for at least 8 years, despite any seasonal and anthropogenic perturbations that have taken place.

Design and development of a PCR primer suite targeting the known diversity of reductive dehalogenase homologous genes provided a mechanism for screening environmental and laboratory samples for members of this gene family. Illumina sequencing of six samples' PCR-amplified products resulted in identification of 798 novel reductive dehalogenase sequences. The newly sequenced reductive dehalogenases helped identify broadly distributed gene “types” whose functions may be important within the natural environment, and who will pose interesting targets for further characterization. The sequenced RdhAs also represent new reference sequences spanning a substantial untapped depth of sequence diversity within the gene family. The distribution of the reductive dehalogenases and their taxonomic affiliations within a contaminated site undergoing bioremediation allowed identification of biomarkers for tracking the enrichment culture used for bioaugmentation. A comparison between a contaminated site and an enrichment culture derived from the same site provided insight into the reduced complexity and community composition changes inherent to the enrichment process.

### Conflict of interest statement

The authors declare that the research was conducted in the absence of any commercial or financial relationships that could be construed as a potential conflict of interest.

## Supplementary material

Data sheet 1: Table S1 **Reductive dehalogenase primer suite summary information.** Expected product size, primer sequences, melting temperature ranges, and degeneracy levels are listed.

Data sheet 2: Table S2 ***rdhA* gene information, including NCBI accessions, best blast matches to the curated RdhA dataset and to NCBI's protein non-redundant database, gene length, and predicted primer group and reductive dehalogenase ortholog groups (RD_OG) for each novel *rdhA***.

Data sheet 3: Table S3 **Reductive dehalogenase homologous genes curated reference dataset information, with tree identifiers linked to NCBI, JGI, or in-house accession numbers and organism of origin**.

Data sheet 4: Table S4 **Companion table to Figure 6, with accession numbers for all sequences included in the abundance table groups, and associated primer groups and RD_OGs for each row**.

Datasheet 5: Figure S1 **Maximum likelihood trees of the reductive dehalogenase primer groups for which newly sequenced RdhA were identified, including the new RdhAs and the reductive dehalogenase curated dataset sequences.** Sequences for each primer group or set of groups were aligned using Muscle v. 3.8.31 (Edgar, 2004), and the alignments manually curated. Trees were generated in PhyML (Guindon and Gascuel, 2003) under the LG + γ model of amino acid evolution. For each alignment, an outgroup RdhA sequence was included to root the trees (not shown). Names for newly sequenced RdhA genes correspond to the contig from the idba_ud assemblies (Peng et al., 2012), with accession numbers in parentheses.
