# Corrigendum: Diversity of reductive dehalogenase genes from environmental samples and enrichment cultures identified with degenerate primer PCR screens

**DOI:** 10.3389/fmicb.2015.00030

**Published:** 2015-02-03

**Authors:** Laura A. Hug, Elizabeth A. Edwards

**Affiliations:** ^1^Department of Cell and Systems Biology, University of TorontoToronto, ON, Canada; ^2^Department of Chemical Engineering and Applied Chemistry, University of TorontoToronto, ON, Canada

**Keywords:** Erratum, Amplicon sequencing, diversity, reductive dehalogenase, organohalide respiration

The PCR amplification conditions were inaccurate as originally published, omitting the number of cycles for the final amplification step—a total of 25 cycles should be conducted when annealing at 50°C. The corrected methods are included below.

The following conditions were subsequently applied to all described samples: an initial denaturation at 95°C for 5 min, three cycles of denaturation at 95°C for 30 s, primer annealing at 38°C for 30 s, and elongation at 72°C for 90 s, three cycles of denaturation at 95°C for 30 s, primer annealing at 45°C for 30 s, and elongation at 72°C for 90 s, and 25 cycles of denaturation at 95°C for 30 s, primer annealing at 50°C for 30 s, and elongation at 72°C for 90 s, and a final extension at 72°C for 10 min.

## Conflict of interest statement

The authors declare that the research was conducted in the absence of any commercial or financial relationships that could be construed as a potential conflict of interest.

