# Supplementary material for: Diversity of reductive dehalogenase genes from environmental samples and enrichment cultures identified with degenerate primer PCR screens
Source: Front Microbiol. 2013 Nov 19;4:341. doi: 10.3389/fmicb.2013.00341 (PMC3832961; doi:10.3389/fmicb.2013.00341)
Supplement: Data sheet 1: Table S1 — Reductive dehalogenase primer suite summary information. Expected product size, primer sequences, melting temperature ranges, and degeneracy levels are listed. [file DataSheet1.DOCX]

Table S1: Reductive dehalogenase primer suite summary information. Expected product size, primer sequences, melting temperature ranges, and degeneracy levels are listed.

|  |  |  | Expected product size | Primer Tm | | |  |
| --- | --- | --- | --- | --- | --- | --- | --- |
| Group | Primer Name | Primer sequence |  | Min | Mean | Max | *Primer degeneracy |
| 1 | RDH_Group1F | TCNMGRCGTGAYTTTATGAA | 1300 | 45.9ºC | 51.6ºC | 57.1ºC | 32 |
|  | RDH_Group1R | ARGTRCCCTGACARGTG |  | 48.2ºC | 52.8ºC | 57.4ºC | 8 |
| 2 | RDH_Group2F | CWGTVCCYATTGACTGGCA | 1220 | 51.4ºC | 55.2ºC | 58.3ºC | 12 |
|  | RDH_Group2R | TCCATATTSGCAAAGAAACTRTTGAA | | 53.2ºC | 54.4ºC | 55.5ºC | 4 |
| 3A&B | RDH_Group3AF | CCCCRGTTTTYCATGAYATGGATGA | 1250 | 55.2ºC | 58.2ºC | 61.2ºC | 8 |
|  | RDH_Group3BF | GGWGCBGGATTDGGTGC | 1290 | 52.7ºC | 56.7ºC | 60.1ºC | 18 |
|  | RDH_Group3R | GGRCARGTACCCTGRCA |  | 50.7ºC | 55.6ºC | 60.5ºC | 8 |
| 4 | RDH_Group4F | CCBKTTTTCCRTGAYDTRGATGAAAT | 1200 | 49.9ºC | 55.8ºC | 61.4ºC | 144 |
|  | RDH_Group4R | TGRCARACYGGRCAATG |  | 46.8ºC | 52.7ºC | 58.7ºC | 16 |
| 5A&B | RDH_Group5AF | GYGGYTGGTRKGTHAARGA | 1020 | 47.0ºC | 54.5ºC | 63.0ºC | 96 |
|  | RDH_Group5BF(3) | CGYSCYTGGTAYRTAAA | 1010 | 42.0ºC | 48.9ºC | 55.3ºC | 32 |
|  | RDH_Group5R | CRCADRTWCCRCARGTYTC |  | 45.6ºC | 53.2ºC | 61.1ºC | 192 |
| 6 | RDH_Group6F | CVGKTTTYCAYGAYYTDGAYGA | 830-900 | 46.7ºC | 55.2ºC | 63.5ºC | 576 |
|  | RDH_Group6R | TGYTCRCCVAYVCCDG |  | 45.2ºC | 55.7ºC | 65.3ºC | 216 |
| 7 | RDH_Group7F | TGGTGGGTDAARSAACG | 1250 | 49.2ºC | 51.9ºC | 55.7ºC | 12 |
|  | RDH_Group7R | TCCATVTBDGCGAARAADC |  | 44.8ºC | 51.6ºC | 58.0ºC | 162 |
| 8 | RDH_Group8F | CGWGATTTYATGAARRGAYTRGG | 1430-1550 | 47.5ºC | 52.5ºC | 57.9ºC | 64 |
|  | RDH_Group8R | AARAAWSHRTTAAAAACVGKYGT |  | 45.1ºC | 51.7ºC | 58.1ºC | 576 |
| 9A | RDH_Group9AF | CCTKGTKGGTGCWGG | 1390 | 50.4ºC | 53.3ºC | 56.2ºC | 8 |
|  | RDH_Group9AR | AGKGTRTCGTRTTYCCA |  | 43.9ºC | 50.0ºC | 56.3ºC | 16 |
| 9B | RDH_Group9BF | TGGTGGGTHAARGARARMGA | 1320 | 49.4ºC | 54.4ºC | 60.7ºC | 48 |
|  | RDH_Group9BR | ARATCYCKRTHCCACCA |  | 43.2ºC | 50.6ºC | 58.5ºC | 48 |
| 10 | RDH_Group10F | GCCGCYYTDCATWMHTWYGG | 800-850 | 49.2ºC | 55.7ºC | 64.2ºC | 576 |
|  | RDH_Group10R | TCCATRKTGGYRAAGAASC |  | 46.5ºC | 52.4ºC | 58.0ºC | 32 |
| 11A | RDH_Group11AF2 | GAATGGAAACGYCCYTGGT | 1200 | 52.7ºC | 55.4ºC | 58.1ºC | 4 |
|  | RDH_Group11AR | CCTGCCACAGGAARCTG |  | 53.4ºC | 54.8ºC | 56.3ºC | 2 |
| 11B | RDH_Group11BF3 | CSGGAGCVGGTATKGGTAC | 1000 | 55.1ºC | 58.0ºC | 60.1ºC | 8 |
| cont’d |  |  | Expected product size | Primer Tm | | |  |
| Group | Primer Name | Primer sequence |  | Min | Mean | Max | *Primer degeneracy |
|  | RDH_Group11BR2 | CATTTDTKGCAGGTWTGGCA |  | 50.8ºC | 53.7ºC | 56.9ºC | 12 |
| 11C | RDH_Group11CF2 | TGGTGGRTBAARKAVCGKGA | 1300 | 49.0ºC | 56.9ºC | 63.5ºC | 108 |
|  | RDH_Group11CR2 | CCRAAGAAYTTRTCHGCCTG |  | 49.0ºC | 53.4ºC | 58.5ºC | 24 |
| 12 | RDH_Group12F3 | CCSTGGTGGRTYAAGGAA | 1180 | 51.2ºC | 54.4ºC | 57.5ºC | 6 |
|  | RDH_Group12R3 | GTATTRAAYACACAAGTACCCATACA | | 50.8ºC | 52.9ºC | 55.0ºC | 4 |
| 13 | RDH_Group13F2 | AAAMGVCCCTGGTGGGT | 1400 | 53.6ºC | 57.6ºC | 60.3ºC | 6 |
|  | RDH_Group13R2 | TACTTTBYGTAYCCGCC |  | 46.3ºC | 50.7ºC | 55.1ºC | 8 |
| 14 | RDH_Group14F2 | GCGTCCCTGGTGGGT | 1120 |  | 58.2ºC |  | 16 |
|  | RDH_Group14R2 | GGYAHATCCCADGARGGTTC |  | 49.7ºC | 54.6ºC | 60.5ºC | 0 |
| 15A | RDH_Group15AF | TGGTGGRTWAARGASGTTGA | 1100 | 51.0ºC | 53.6ºC | 56.2ºC | 16 |
|  | RDH_Group15AR | GTCATGWATACYKGCTTTRTYYTTCT | | 50.2ºC | 54.3ºC | 58.9ºC | 64 |
| 15B | RDH_group15BF | AAGGCAMMCCYGARGARAA | 860 | 49.9ºC | 55.5ºC | 61.3ºC | 32 |
|  | RDH_Group15BR | TAVCCRAAKAARTCAKMCA |  | 40.7ºC | 48.5ºC | 55.9ºC | 96 |
| 16 | RDH_Group16F | CCBTGGTRKGTNMRWGA | 1200-1300 | 42.5ºC | 51.6ºC | 61.2ºC | 384 |
|  | RDH_Group16R | CCAAAKSCWYKRTCCATATT |  | 44.9ºC | 50.2ºC | 55.4ºC | 32 |
| 17 | RDH_Group17F | GCARTTGAAAAAYTRCCATGGTG | 1100 | 51.5ºC | 54.5ºC | 57.6ºC | 8 |
|  | RDH_Group17R | GCTATCATTAGCATCMCGMCC |  | 53.3ºC | 55.6ºC | 58.1ºC | 4 |
| 18A | RDH_Group18AF | CACAACACKYTATCMAGRCGG | 1080 | 51.6ºC | 56.0ºC | 60.8ºC | 16 |
|  | RDH_Group18RA | GGGRCAKKTATCRGMRCATTT |  | 48.3ºC | 55.5ºC | 62.9ºC | 64 |
| 18B | RDH_group18BF2 | ACARTRAGYMGMMGAGATTTTATGAA | 1000 | 48.5ºC | 54.6ºC | 60.4ºC | 64 |
|  | RDH_group18BR2 | GGCAAATCDGTVARSAKAA |  | 44.7ºC | 50.2ºC | 55.8ºC | 48 |
| 19 | RDH_Group19F | CMGMMGRGATTTYATGAARR | 1260 | 43.0ºC | 50.7ºC | 58.8ºC | 128 |
|  | RDH_Group19R | TCCATRYTYYTGAAGAARCYGTT |  | 48.8ºC | 54.6ºC | 60.8ºC | 64 |
| 20 | RDH_Group20F | GCCTGAACTCTCAAGAAGAG | 1470 |  | 52.6 |  | 0 |
|  | RDH_Group20R | GCTTACAGTGTCTCTAAAATGGTC | |  | 53.5 |  | 0 |
| 21 | RDH_Group21F3 | TCCGYWMCCTYGGCTA | 640 | 49.3ºC | 54.1ºC | 59.1ºC | 16 |
|  | RDH_Group21R3 | ATGCAGWTSGCGCAA |  | 52.0ºC | 52.3ºC | 52.6ºC | 4 |
| 22 | RDH_Group22F | AAYGAYSARTGGYTBGGNAC | 1100 | 48.2ºC | 55.8ºC | 63.0ºC | 384 |
|  | RDH_Group22R | GCCAYTCYARCCACCAYTT |  | 50.4ºC | 55.5ºC | 60.6ºC | 16 |
| 22B | RDH_Group22BF | TTGTRGACGGRCCKGTAAGA | 870 | 52.4ºC | 56.8ºC | 61.2ºC | 8 |
| cont’d |  |  | Expected product size | Primer Tm | | |  |
| Group | Primer Name | Primer sequence |  | Min | Mean | Max | *Primer degeneracy |
|  | RDH_Group22BR | GGTACCRTAWCCRAAGAAATCATC | | 51.5ºC | 53.4ºC | 55.4ºC | 8 |
| 23 | RDH_Group23F | ATGRCCTCGGCSGC | 1100 | 55.4ºC | 57.5ºC | 59.7ºC | 4 |
|  | RDH_Group23R | CGCKMGACGCCGC |  | 53.5ºC | 58.2ºC | 63.0ºC | 4 |
| 24 | RDH_Group24F | CTRSKTWYAATGABTATTATWMGAAAAATCC | 850 | 48.1ºC | 52.5ºC | 56.7ºC | 384 |
|  | RDH_Group24R | CACMTACCACAATCKGTWCC |  | 50.4ºC | 53.0ºC | 55.5ºC | 8 |
| 25 | RDH_Group25F | TKGAYGCMMGBSAACATGG | 1000 | 50.9ºC | 57.7ºC | 63.8ºC | 96 |
|  | RDH_Group25R | GTRTCHGGTTTDGACCAVGG |  | 49.9ºC | 55.2ºC | 61.1ºC | 54 |
| 26 | RDH_Group26F2 | CARGAAMAYCGYCARGGT | 1380 | 47.4ºC | 53.9ºC | 60.5ºC | 32 |
|  | RDH_Group26R2 | GGAGGKTCRTARACACC |  | 47.0ºC | 50.6ºC | 54.3ºC | 8 |
| 27 | RDH_Group27F | GASWGSGAYTTTMAGGGYGTA | 570 | 51.4ºC | 55.6ºC | 59.5ºC | 64 |
|  | RDH_Group27R | GGCTCYAWAGGMAKRTTSGTC |  | 49.6ºC | 55.4ºC | 61.1ºC | 64 |
| 28 | RDH_Group28F | GRAKATSGCKGMCTGRTMA | 930-970 | 46.4ºC | 54.4ºC | 62.6ºC | 128 |
|  | RDH_Group28R | TSGRAMRRKGWTYTYCTCT |  | 44.3ºC | 51.8ºC | 60.4ºC | 512 |
| 29 | RDH_Group29F2 | GCKMACCYKCTCCG | 780 | 44.9ºC | 52.9ºC | 61.3ºC | 16 |
|  | RDH_Group29R | CYSGYCSACCTGSYAG |  | 51.3ºC | 57.1ºC | 62.9ºC | 64 |
| 30 | RDH_Group30F | GRMGMCTCYGGRAGWAGC | 815 | 49.1ºC | 56.5ºC | 64.4ºC | 64 |
|  | RDH_Group30R | GTWTTKGGYTTRTTRTAVGGACAA | | 47.0ºC | 52.9ºC | 58.3ºC | 96 |
| 31A | RDH_Group31AF | CAGCTCTCACATCAGTTGTAGC | 1170 |  | 55.5ºC |  | 0 |
| 31B | RDH_group31BF | TGGATAAAACAMARAGTYGAAGG | 960 | 49.9ºC | 52.3ºC | 54.6ºC | 8 |
|  | RDH_Group31R | GRCAWGMHTCRGCRCA |  | 45.9ºC | 53.9ºC | 62.8ºC | 96 |
| 32 | RDH_Group32F3 | GACGATGTTTCCAARATCACMGG | 1350 | 54.5ºC | 56.2ºC | 58.0ºC | 4 |
|  | RDH_Group32R | TTCCAGAARTCTKTKACCATGTT |  | 50.6ºC | 53.4ºC | 56.2ºC | 8 |
| 33 | RDH_group33F | GATTGTAGAAGCAGCGG | 1500 |  | 50.5ºC |  | 0 |
|  | RDH_group33R | CCACCAATCTGCTATCGC |  |  | 53.4ºC |  | 0 |
| 34 | RDH_group34F | GGARTRGGGGYYGG | 1200 | 44.4ºC | 51.8ºC | 59.6ºC | 16 |
|  | RDH_group34R | CCRAACARATCRTCAAAGAACCT |  | 52.0ºC | 54.5ºC | 57.0ºC | 8 |

*Primer degeneracy was calculated by multiplying the individual degeneracies of each primer position together. eg., for sequence “CYN”, position degeneracies are 1, 2, and 4 respectively, meaning the total degeneracy is 1x2x4 = 8
