# Supplementary material for: Diversity of reductive dehalogenase genes from environmental samples and enrichment cultures identified with degenerate primer PCR screens
Source: Front Microbiol. 2013 Nov 19;4:341. doi: 10.3389/fmicb.2013.00341 (PMC3832961; doi:10.3389/fmicb.2013.00341)
Supplement: Data sheet 3: Table S3 — Reductive dehalogenase homologous genes curated reference dataset information, with tree identifiers linked to NCBI, JGI, or in-house accession numbers and organism of origin. [file DataSheet3.DOCX]

**Table S3:** Reductive dehalogenase homologous genes curated dataset information, with tree identifiers linked to NCBI, JGI, or in-house accession numbers and organism of origin. A version of this table appeared as Table S1 in Hug et al. (2013).

| **NAME ON TREE** | **ORGANISM** | **KEY** | **WHAT KEY IS** |
| --- | --- | --- | --- |
| A2cp1_0353 | *Anaeromyxobacter dehalogenans* 2CP-1 | A2cp1_0353 | JGI locus tag |
| A2cp1_0355 | *Anaeromyxobacter dehalogenans* 2CP-1 | A2cp1_0355 | JGI locus tag |
| AB194705 | *Desulfitobacterium* sp. KBC1 | AB194705 | NCBI Accession |
| AB194706 | *Desulfitobacterium* sp. KBC1 (*prdA*) | AB194706 | NCBI Accession |
| ACP_0433 | *Acidobacterium capsulatum* | ACP_0433 | JGI locus tag |
| ACT3-rdh01 | *Dehalobacter* CF (*cfrA*) | AFV05253 | NCBI Accession |
| ACT3-rdh02 | *Dehalobacter* DCA (*dcrA*) | AFV02209 | NCBI Accession |
| ACT3-rdh03 | *Dehalobacter* CF | AFV06361 | NCBI Accession |
| ACT3-rdh04 | *Dehalobacter* CF | AFV06212 | NCBI Accession |
| ACT3-rdh05 | *Dehalobacter* CF | AFV06391 | NCBI Accession |
| ACT3-rdh06 | *Dehalobacter* CF | AFV05217 | NCBI Accession |
| ACT3-rdh07 | *Dehalobacter* CF | AFV05214 | NCBI Accession |
| ACT3-rdh08 | *Dehalobacter* CF | AFV05205 | NCBI Accession |
| ACT3-rdh09 | *Dehalobacter* DCA | AFV02160 | NCBI Accession |
| ACT3-rdh10 | *Dehalobacter* CF | AFV05983 | NCBI Accession |
| ACT3-rdh11 | *Dehalobacter* CF | AFV05981 | NCBI Accession |
| ACT3-rdh12 | *Dehalobacter* CF | AFV05683 | NCBI Accession |
| ACT3-rdh13 | *Dehalobacter* CF | AFV05674 | NCBI Accession |
| ACT3-rdh14 | *Dehalobacter* CF | AFV04610 | NCBI Accession |
| ACT3-rdh15 | *Dehalobacter* CF | AFV06370 | NCBI Accession |
| ACT3-rdh16 | *Dehalobacter* CF | AFV06369 | NCBI Accession |
| ACT3-rdh17 | *Dehalobacter* CF | AFV06381 | NCBI Accession |
| ACT3-rdh18 | *Dehalobacter* CF | AFV06387 | NCBI Accession |
| ACT3-rdh19 | *Dehalobacter* CF | AFV06377 | NCBI Accession |
| Adeh_0329 | *Anaeromyxobacter dehalogenans* 2CP-C | 3886157 | NCBI Gene ID |
| Adeh_0331 | *Anaeromyxobacter dehalogenans* 2CP-C | 3886159 | NCBI Gene ID |
| AF022812 | *Sulfurospirillum multivorans* | AF022812 | NCBI Accession |
| AF115542 | *Desulfitobacterium dehalogenans* ATCC 51507 (*cprA*) | AF115542 | NCBI Accession |
| AF204275 | *Desulfitobacterium chlororespirans* | AF204275 | NCBI Accession |
| AF259790 | *Desulfitobacterium* sp. PCE1 | AF259790 | NCBI Accession |
| AF259791 | *Desulfitobacterium* sp. Viet-1 | AF259791 | NCBI Accession |
| AGWLrdhA1 | *Dehalobacter* WL (*dcrA*) | FJ010189 | NCBI Accession |
| AJ439607 | *Dehalobacter restrictus* PER-K23 (*pceA*) | AJ439607 | NCBI Accession |
| AJ539533 | *Dehalobacter restrictus* PER-K23 | AJ539533 | NCBI Accession |
|  |  |  |  |
| **NAME ON TREE** | **ORGANISM** | **KEY** | **WHAT KEY IS** |
| AM183918 | *Desulfitobacterium dichloroeliminans* LMG P-21439 (*dcaA*) | AM183918 | NCBI Accession |
| AnaeK_0341 | *Anaeromyxobacter* *dehalogenans* K | 6786349 | NCBI Gene ID |
| AnaeK_0343 | *Anaeromyxobacter* *dehalogenans* K | 6786351 | NCBI Gene ID |
| AY013360 | *Desulfitobacterium* sp. PCE1 | AY013360 | NCBI Accession |
| AY013361 | *Desulfitobacterium* sp. PCE1 | AY013361 | NCBI Accession |
| AY013362 | *Desulfitobacterium hafniense* TCE1 | AY013362 | NCBI Accession |
| AY013363 | *Desulfitobacterium dehalogenans* | AY013363 | NCBI Accession |
| AY013364 | *Desulfitobacterium hafniense* PCP-1 | AY013364 | NCBI Accession |
| AY013365 | *Desulfitobacterium hafniense* DCB-2 | AY013365 | NCBI Accession |
| AY013366 | *Desulfitobacterium hafniense* DCB-2 | AY013366 | NCBI Accession |
| AY013367 | *Sulfurospirillum halorespirans* PCE-M2 | AY013367 | NCBI Accession |
| AY165309 | *Dehalococcoides* *mccartyi* FL2 | AY165309 | NCBI Accession |
| AY216592 | *Desulfitobacterium* sp. PCE-S | AY216592 | NCBI Accession |
| AY349165 | *Desulfitobacterium hafniense* PCP-1 (*cprA*) | AY349165 | NCBI Accession |
| AY374245 | *Dehalococcoides* *mccartyi* FL2 | AY374245 | NCBI Accession |
| AY374246 | *Dehalococcoides* *mccartyi* FL2 | AY374246 | NCBI Accession |
| AY374247 | *Dehalococcoides* *mccartyi* FL2 | AY374247 | NCBI Accession |
| AY374248 | *Dehalococcoides* *mccartyi* FL2 | AY374248 | NCBI Accession |
| AY374249 | *Dehalococcoides* *mccartyi* FL2 | AY374249 | NCBI Accession |
| AY374250 | *Dehalococcoides* *mccartyi* FL2 | AY374250 | NCBI Accession |
| AY374251 | *Dehalococcoides* *mccartyi* FL2 | AY374251 | NCBI Accession |
| AY374252 | *Dehalococcoides* *mccartyi* FL2 | AY374252 | NCBI Accession |
| AY374253 | *Dehalococcoides* *mccartyi* FL2 | AY374253 | NCBI Accession |
| AY374254 | *Dehalococcoides* *mccartyi* FL2 | AY374254 | NCBI Accession |
| BAV1_0104 | *Dehalococcoides* *mccartyi* BAV1 | 5131704 | NCBI Gene ID |
| BAV1_0112 | *Dehalococcoides* *mccartyi* BAV1 | 5132438 | NCBI Gene ID |
| BAV1_0119 | *Dehalococcoides* *mccartyi* BAV1 | 5131444 | NCBI Gene ID |
| BAV1_0121 | *Dehalococcoides* *mccartyi* BAV1 | 5131472 | NCBI Gene ID |
| BAV1_0173 | *Dehalococcoides* *mccartyi* BAV1 | 5132211 | NCBI Gene ID |
| BAV1_0276 | *Dehalococcoides* *mccartyi* BAV1 (*bvcA*) | 5132094 | NCBI Gene ID |
| BAV1_0281 | *Dehalococcoides* *mccartyi* BAV1 | 5132381 | NCBI Gene ID |
| BAV1_0284 | *Dehalococcoides* *mccartyi* BAV1 | 5132431 | NCBI Gene ID |
| BAV1_0296 | *Dehalococcoides* *mccartyi* BAV1 | 5132361 | NCBI Gene ID |
| BAV1_0847 | *Dehalococcoides* *mccartyi* BAV1 | 5131305 | NCBI Gene ID |
| cbdbA1092 | *Dehalococcoides* *mccartyi* CBDB1 | 3623213 | NCBI Gene ID |
| cbdbA1453 | *Dehalococcoides* *mccartyi* CBDB1 | 3623214 | NCBI Gene ID |
| cbdbA1455 | *Dehalococcoides* *mccartyi* CBDB1 | 3623215 | NCBI Gene ID |
| cbdbA1491 | *Dehalococcoides* *mccartyi* CBDB1 | 3623216 | NCBI Gene ID |
| **NAME ON TREE** | **ORGANISM** | **KEY** | **WHAT KEY IS** |
| cbdbA1495 | *Dehalococcoides* *mccartyi* CBDB1 | 3623217 | NCBI Gene ID |
| cbdbA1503 | *Dehalococcoides* *mccartyi* CBDB1 | 3623218 | NCBI Gene ID |
| cbdbA1508 | *Dehalococcoides* *mccartyi* CBDB1 | 3623219 | NCBI Gene ID |
| cbdbA1535 | *Dehalococcoides* *mccartyi* CBDB1 | 3623220 | NCBI Gene ID |
| cbdbA1539 | *Dehalococcoides* *mccartyi* CBDB1 | 3623221 | NCBI Gene ID |
| cbdbA1542 | *Dehalococcoides* *mccartyi* CBDB1 | 3623222 | NCBI Gene ID |
| cbdbA1546 | *Dehalococcoides* *mccartyi* CBDB1 | 3623223 | NCBI Gene ID |
| cbdbA1550 | *Dehalococcoides* *mccartyi* CBDB1 | 3623444 | NCBI Gene ID |
| cbdbA1560 | *Dehalococcoides* *mccartyi* CBDB1 | 3623445 | NCBI Gene ID |
| cbdbA1563 | *Dehalococcoides* *mccartyi* CBDB1 | 3623446 | NCBI Gene ID |
| cbdbA1570 | *Dehalococcoides* *mccartyi* CBDB1 | 3623447 | NCBI Gene ID |
| cbdbA1575 | *Dehalococcoides* *mccartyi* CBDB1 | 3623448 | NCBI Gene ID |
| cbdbA1578 | *Dehalococcoides* *mccartyi* CBDB1 | 3623468 | NCBI Gene ID |
| cbdbA1582 | *Dehalococcoides* *mccartyi* CBDB1 | 3623469 | NCBI Gene ID |
| cbdbA1588 | *Dehalococcoides* *mccartyi* CBDB1 | 3623470 | NCBI Gene ID |
| cbdbA1595 | *Dehalococcoides* *mccartyi* CBDB1 | 3623471 | NCBI Gene ID |
| cbdbA1598 | *Dehalococcoides* *mccartyi* CBDB1 | 3623472 | NCBI Gene ID |
| cbdbA1618 | *Dehalococcoides* *mccartyi* CBDB1 | 3623473 | NCBI Gene ID |
| cbdbA1624 | *Dehalococcoides* *mccartyi* CBDB1 | 3623474 | NCBI Gene ID |
| cbdbA1627 | *Dehalococcoides* *mccartyi* CBDB1 | 3623475 | NCBI Gene ID |
| cbdbA1638 | *Dehalococcoides* *mccartyi* CBDB1 | 3623476 | NCBI Gene ID |
| cbdbA187 | *Dehalococcoides* *mccartyi* CBDB1 | 3623477 | NCBI Gene ID |
| cbdbA238 | *Dehalococcoides* *mccartyi* CBDB1 | 3623478 | NCBI Gene ID |
| cbdbA243 | *Dehalococcoides* *mccartyi* CBDB1 | 3623479 | NCBI Gene ID |
| cbdbA80 | *Dehalococcoides* *mccartyi* CBDB1 | 3623480 | NCBI Gene ID |
| cbdbA84 | *Dehalococcoides* *mccartyi* CBDB1 *(cbrA*) | 3623481 | NCBI Gene ID |
| cbdbA88 | *Dehalococcoides* *mccartyi* CBDB1 | 3623482 | NCBI Gene ID |
| cbdbA96 | *Dehalococcoides* *mccartyi* CBDB1 | 3623483 | NCBI Gene ID |
| CD1958 * | *Clostridium difficile* 630 | 115249003 | NCBI Gene ID |
| CD196_1838 * | *Clostridium difficile* R20291 | 260211391 | NCBI Gene ID |
| CR1_tceA | *Desulfitobacterium* sp. CR1 | 145860191 | NCBI Gene ID |
| D328-MS | *Dehalobacter* MS (MS_rdhA1) | FJ010192 | NCBI Accession |
| D329-MS | *Dehalobacter* MS (MS_rdhA2) | FJ010193 | NCBI Accession |
| D333-WLm | *Dehalobacter* WL (WL_rdhA2) | FJ010190 | NCBI Accession |
| D334-WLm | *Dehalobacter* WL (WL_rdhA3) | FJ010191 | NCBI Accession |
| D335-WL | *Dehalococcoides* *mccartyi* WL |  | in house name |
| D336-WL | *Dehalococcoides* *mccartyi* WL |  | in house name |
| D337-WL | *Dehalococcoides* *mccartyi* WL |  | in house name |
| D338-WL | *Dehalococcoides* *mccartyi* WL | (WL_*Dhc_*01) | in house name |
| D339-WL | *Dehalococcoides* *mccartyi* WL |  | in house name |
| DealDRAFT_0257 | *Dethiobacter alkaliphilus* AHT 1 | DealDRAFT_0257 | JGI locus tag |
| Dehly0068 | *Dehalogenimonas lykanthroporepellens* BL-DC-9 | 9389745 | NCBI Gene ID |
|  |  |  |  |
|  |  |  |  |
| **NAME ON TREE** | **ORGANISM** | **KEY** | **WHAT KEY IS** |
| Dehly0121 | *Dehalogenimonas lykanthroporepellens* BL-DC-9 | 9389797 | NCBI Gene ID |
| Dehly0156 | *Dehalogenimonas lykanthroporepellens* BL-DC-9 | 9389835 | NCBI Gene ID |
| Dehly0274 | *Dehalogenimonas lykanthroporepellens* BL-DC-9 | 9389954 | NCBI Gene ID |
| Dehly0275 | *Dehalogenimonas lykanthroporepellens* BL-DC-9 | 9389955 | NCBI Gene ID |
| Dehly0283 | *Dehalogenimonas lykanthroporepellens* BL-DC-9 | 9389963 | NCBI Gene ID |
| Dehly0849 | *Dehalogenimonas lykanthroporepellens* BL-DC-9 | 9390556 | NCBI Gene ID |
| Dehly0910 | *Dehalogenimonas lykanthroporepellens* BL-DC-9 | 9390618 | NCBI Gene ID |
| Dehly1054 | *Dehalogenimonas lykanthroporepellens* BL-DC-9 | 9390763 | NCBI Gene ID |
| Dehly1148 | *Dehalogenimonas lykanthroporepellens* BL-DC-9 | 9390857 | NCBI Gene ID |
| Dehly1152 | *Dehalogenimonas lykanthroporepellens* BL-DC-9 | 9390861 | NCBI Gene ID |
| Dehly1328 | *Dehalogenimonas lykanthroporepellens* BL-DC-9 | 9391042 | NCBI Gene ID |
| Dehly1355 | *Dehalogenimonas lykanthroporepellens* BL-DC-9 | 9391069 | NCBI Gene ID |
| Dehly1514 | *Dehalogenimonas lykanthroporepellens* BL-DC-9 | 9391237 | NCBI Gene ID |
| Dehly1520 | *Dehalogenimonas lykanthroporepellens* BL-DC-9 | 9391243 | NCBI Gene ID |
| Dehly1524 | *Dehalogenimonas lykanthroporepellens* BL-DC-9 | 9391247 | NCBI Gene ID |
| Dehly1530 | *Dehalogenimonas lykanthroporepellens* BL-DC-9 | 9391253 | NCBI Gene ID |
| Dehly1540 | *Dehalogenimonas lykanthroporepellens* BL-DC-9 | 9391263 | NCBI Gene ID |
| Dehly1582 | *Dehalogenimonas lykanthroporepellens* st. BL-DC-9 | 9391305 | NCBI Gene ID |
| DET0079_tceA | *Dehalococcoides mccartyi* 195 (*tceA*) | 3229017 | NCBI Gene ID |
| DET0088 | *Dehalococcoides mccartyi* 195 | 3229009 | NCBI Gene ID |
| DET0173 | *Dehalococcoides mccartyi* 195 | 3230555 | NCBI Gene ID |
| DET0180 | *Dehalococcoides mccartyi* 195 | 3230541 | NCBI Gene ID |
| DET0235 | *Dehalococcoides mccartyi* 195 | 3230432 | NCBI Gene ID |
| DET0302 | *Dehalococcoides mccartyi* 195 | 3230337 | NCBI Gene ID |
| DET0306 | *Dehalococcoides mccartyi* 195 | 3230325 | NCBI Gene ID |
| DET0311 | *Dehalococcoides mccartyi* 195 | 3230320 | NCBI Gene ID |
| DET0318 | *Dehalococcoides mccartyi* 195 (*pceA*) | 3230306 | NCBI Gene ID |
| DET0876 | *Dehalococcoides mccartyi* 195 | 3229805 | NCBI Gene ID |
| DET1171 | *Dehalococcoides mccartyi* 195 | 3229566 | NCBI Gene ID |
| DET1519 | *Dehalococcoides mccartyi* 195 | 3229231 | NCBI Gene ID |
| **NAME ON TREE** | **ORGANISM** | **KEY** | **WHAT KEY IS** |
| DET1522 | *Dehalococcoides mccartyi* 195 | 3229220 | NCBI Gene ID |
| DET1528 | *Dehalococcoides mccartyi* 195 | 3229214 | NCBI Gene ID |
| DET1535 | *Dehalococcoides mccartyi* 195 | 3229198 | NCBI Gene ID |
| DET1538 | *Dehalococcoides mccartyi* 195 | 3229187 | NCBI Gene ID |
| DET1545 | *Dehalococcoides mccartyi* 195 | 3229172 | NCBI Gene ID |
| DET1559 | *Dehalococcoides mccartyi* 195 | 3229173 | NCBI Gene ID |
| Dhaf_0689 | *Desulfitobacterium hafniense* DCB-2 | 7257656 | NCBI Gene ID |
| Dhaf_0693 | *Desulfitobacterium hafniense* DCB-2 | 7257660 | NCBI Gene ID |
| Dhaf_0696 | *Desulfitobacterium hafniense* DCB-2 | 7257663 | NCBI Gene ID |
| Dhaf_0711 | *Desulfitobacterium hafniense* DCB-2 | 7257678 | NCBI Gene ID |
| Dhaf_0713 | *Desulfitobacterium hafniense* DCB-2 | 7257680 | NCBI Gene ID |
| Dhaf_0737 | *Desulfitobacterium hafniense* DCB-2 | 7257704 | NCBI Gene ID |
| Dhaf_2620 | *Desulfitobacterium hafniense* DCB-2 | 7259622 | NCBI Gene ID |
| Dhaf_pceA | *Desulfitobacterium hafniense* Y51 | 89332194 | NCBI Gene ID |
| DQ115513 | *Dehalococcoides* *mccartyi* FL2 | 73913555 | NCBI Gene ID |
| DQ115514 | *Dehalococcoides* *mccartyi* FL2 | 73913558 | NCBI Gene ID |
| Ferp_2321 | *Ferroglobus placidus* DSM 10642 | 8779861 | NCBI Gene ID |
| Glov_2870 | *Geobacter lovleyi* SZ | 6369093 | NCBI Gene ID |
| Glov_2872 | *Geobacter lovleyi* SZ | 6366908 | NCBI Gene ID |
| GT_0124 | *Dehalococcoides* *mccartyi* GT | 8808573 | NCBI Gene ID |
| GT_0241 | *Dehalococcoides* *mccartyi* GT | 8808689 | NCBI Gene ID |
| GT_1189 | *Dehalococcoides* *mccartyi* GT | 8809639 | NCBI Gene ID |
| GT_1191 | *Dehalococcoides* *mccartyi* GT | 8809641 | NCBI Gene ID |
| GT_1237 | *Dehalococcoides* *mccartyi* GT | 8809688 | NCBI Gene ID |
| GT_1269 | *Dehalococcoides* *mccartyi* GT | 8809720 | NCBI Gene ID |
| GT_1276 | *Dehalococcoides* *mccartyi* GT | 8809727 | NCBI Gene ID |
| GT_1285 | *Dehalococcoides* *mccartyi* GT | 8809736 | NCBI Gene ID |
| GT_1287 | *Dehalococcoides* *mccartyi* GT | 8809738 | NCBI Gene ID |
| GT_1295 | *Dehalococcoides* *mccartyi* GT | 8809746 | NCBI Gene ID |
| GT_1300 | *Dehalococcoides* *mccartyi* GT | 8809751 | NCBI Gene ID |
| GT_1303 | *Dehalococcoides* *mccartyi* GT | 8809754 | NCBI Gene ID |
| GT_1307 | *Dehalococcoides* *mccartyi* GT | 8809758 | NCBI Gene ID |
| GT_1312 | *Dehalococcoides* *mccartyi* GT | 8809763 | NCBI Gene ID |
| GT_1318 | *Dehalococcoides* *mccartyi* GT | 8809769 | NCBI Gene ID |
| GT_1321 | *Dehalococcoides* *mccartyi* GT | 8809772 | NCBI Gene ID |
| GT_1338 | *Dehalococcoides* *mccartyi* GT | 8809789 | NCBI Gene ID |
| GT_1344 | *Dehalococcoides* *mccartyi* GT | 8809795 | NCBI Gene ID |
| GT_1347 | *Dehalococcoides* *mccartyi* GT | 8809798 | NCBI Gene ID |
| GT_1353 | *Dehalococcoides* *mccartyi* GT | 8809804 | NCBI Gene ID |
| HeliMode_  pceA | *Heliobacterium modesticaldum* Ice1 | 171696369 | NCBI Gene ID |
| Jann_1968 | *Jannaschia* sp. CCS1 | 3934419 | NCBI Gene ID |
| **NAME ON TREE** | **ORGANISM** | **KEY** | **WHAT KEY IS** |
|  |  |  |  |
|  |  |  |  |
| KB1_1 | KB-1 consortium | 77176847 | NCBI Gene ID |
| KB1_10 | KB-1 consortium | 77176874 | NCBI Gene ID |
| KB1_11 | KB-1 consortium | 77176877 | NCBI Gene ID |
| KB1_12 | KB-1 consortium | 77176880 | NCBI Gene ID |
| KB1_13 | KB-1 consortium | 77176883 | NCBI Gene ID |
| KB1_14 | KB-1 consortium | 77176886 | NCBI Gene ID |
| KB1_2 | KB-1 consortium | 77176850 | NCBI Gene ID |
| KB1_22 | KB-1 consortium | JX081249 | NCBI Accession |
| KB1_3 | KB-1 consortium | 77176853 | NCBI Gene ID |
| KB1_4 | KB-1 consortium | 77176856 | NCBI Gene ID |
| KB1_5 | KB-1 consortium | 77176859 | NCBI Gene ID |
| KB1_6 | KB-1 consortium | 77176862 | NCBI Gene ID |
| KB1_7 | KB-1 consortium | 77176865 | NCBI Gene ID |
| KB1_8 | KB-1 consortium | 77176868 | NCBI Gene ID |
| KB1_9 | KB-1 consortium | 77176871 | NCBI Gene ID |
| KB11024_1 | KB-1 consortium | DCKB1_37290 | JGI locus tag |
| KB13107_1 | KB-1 consortium | DCKB1_107470 | JGI locus tag |
| KB13107_2 | KB-1 consortium | DCKB1_107520 | JGI locus tag |
| KB13108_1 | KB-1 consortium | DCKB1_107910 | JGI locus tag |
| KB13109_4 | KB-1 consortium | DCKB1_110270 | JGI locus tag |
| KB13109_7 | KB-1 consortium | DCKB1_110600 | JGI locus tag |
| KB13109_9 | KB-1 consortium | DCKB1_110480 | JGI locus tag |
| KB13240_1 | KB-1 consortium | DCKB1_114790 | JGI locus tag |
| KB13241_1 | KB-1 consortium | DCKB1_115000 | JGI locus tag |
| KB13241_2 | KB-1 consortium | DCKB1_115150 | JGI locus tag |
| KB13241_3 | KB-1 consortium | DCKB1_115210 | JGI locus tag |
| KB13241_4 | KB-1 consortium | DCKB1_115020 | JGI locus tag |
| KB13241_5 | KB-1 consortium | DCKB1_115090 | JGI locus tag |
| KB13241_6 | KB-1 consortium | DCKB1_114910 | JGI locus tag |
| KB13241_7 | KB-1 consortium | DCKB1_114830 | JGI locus tag |
| KB13241_8 | KB-1 consortium | DCKB1_114860 | JGI locus tag |
| KB1338_1 | KB-1 consortium | DCKB1_14890 | JGI locus tag |
| SPO1738 | *Ruegeria pomeroyi* DSS-3 | 3192799 | NCBI Gene ID |
| Ssed1729 | *Shewanella sediminis* HAW-EB3 | 5610108 | NCBI Gene ID |
| Ssed2100 | *Shewanella sediminis* HAW-EB3 | 5612296 | NCBI Gene ID |
| 8657036VS | *Dehalococcoides* *mccartyi* VS | 8657036 | NCBI Gene ID |
| 8657042VS | *Dehalococcoides* *mccartyi* VS | 8657042 | NCBI Gene ID |
| 8657050VS | *Dehalococcoides* *mccartyi* VS | 8657050 | NCBI Gene ID |
| 8657053VS | *Dehalococcoides* *mccartyi* VS | 8657053 | NCBI Gene ID |
| 8657058VS | *Dehalococcoides* *mccartyi* VS | 8657058 | NCBI Gene ID |
| 8657123VS | *Dehalococcoides* *mccartyi* VS | 8657123 | NCBI Gene ID |
| 8658187VS | *Dehalococcoides* *mccartyi* VS | 8658187 | NCBI Gene ID |
| 8658189VS | *Dehalococcoides* *mccartyi* VS | 8658189 | NCBI Gene ID |
| 8658217VS | *Dehalococcoides* *mccartyi* VS (*vcrA*) | 8658217 | NCBI Gene ID |
| **NAME ON TREE** | **ORGANISM** | **KEY** | **WHAT KEY IS** |
| 8658233VS | *Dehalococcoides* *mccartyi* VS | 8658233 | NCBI Gene ID |
| 8658239VS | *Dehalococcoides* *mccartyi* VS | 8658239 | NCBI Gene ID |
| 8658241VS | *Dehalococcoides* *mccartyi* VS | 8658241 | NCBI Gene ID |
| 8658245VS | *Dehalococcoides* *mccartyi* VS | 8658245 | NCBI Gene ID |
| 8658249VS | *Dehalococcoides* *mccartyi* VS | 8658249 | NCBI Gene ID |
| 8658252VS | *Dehalococcoides* *mccartyi* VS | 8658252 | NCBI Gene ID |
| 8658254VS | *Dehalococcoides* *mccartyi* VS | 8658254 | NCBI Gene ID |
| 8658261VS | *Dehalococcoides* *mccartyi* VS | 8658261 | NCBI Gene ID |
| 8658265VS | *Dehalococcoides* *mccartyi* VS | 8658265 | NCBI Gene ID |
| 8658267VS | *Dehalococcoides* *mccartyi* VS | 8658267 | NCBI Gene ID |
| 8658269VS | *Dehalococcoides* *mccartyi* VS | 8658269 | NCBI Gene ID |
| 8658272VS | *Dehalococcoides* *mccartyi* VS | 8658272 | NCBI Gene ID |
| 8658274VS | *Dehalococcoides* *mccartyi* VS | 8658274 | NCBI Gene ID |
| 8658278VS | *Dehalococcoides* *mccartyi* VS | 8658278 | NCBI Gene ID |
| 8658285VS | *Dehalococcoides* *mccartyi* VS | 8658285 | NCBI Gene ID |
| 8658289VS | *Dehalococcoides* *mccartyi* VS | 8658289 | NCBI Gene ID |
| 8658296VS | *Dehalococcoides* *mccartyi* VS | 8658296 | NCBI Gene ID |
| 8658300VS | *Dehalococcoides* *mccartyi* VS | 8658300 | NCBI Gene ID |
| 8658303VS | *Dehalococcoides* *mccartyi* VS | 8658303 | NCBI Gene ID |
| 8658308VS | *Dehalococcoides* *mccartyi* VS | 8658308 | NCBI Gene ID |
| 8658312VS | *Dehalococcoides* *mccartyi* VS | 8658312 | NCBI Gene ID |
| 8658318VS | *Dehalococcoides* *mccartyi* VS | 8658318 | NCBI Gene ID |
| 8658324VS | *Dehalococcoides* *mccartyi* VS | 8658324 | NCBI Gene ID |
| 8658327VS | *Dehalococcoides* *mccartyi* VS | 8658327 | NCBI Gene ID |
| 8658346VS | *Dehalococcoides* *mccartyi* VS | 8658346 | NCBI Gene ID |
| 8658352VS | *Dehalococcoides* *mccartyi* VS | 8658352 | NCBI Gene ID |
| 8658355VS | *Dehalococcoides* *mccartyi* VS | 8658355 | NCBI Gene ID |
| 8658361VS | *Dehalococcoides* *mccartyi* VS | 8658361 | NCBI Gene ID |

* = RdhA sequences from *Clostridium* spp. contain the C-terminal portion of the reductive dehalogenase domain as well as the FeS-binding motif, but do not contain the Tat signal sequence or N-terminal portion of the RDase domain. They have been included here barring evidence they do not function as reductive dehalogenases.
