# Supplementary material for: Diversity of reductive dehalogenase genes from environmental samples and enrichment cultures identified with degenerate primer PCR screens
Source: Front Microbiol. 2013 Nov 19;4:341. doi: 10.3389/fmicb.2013.00341 (PMC3832961; doi:10.3389/fmicb.2013.00341)
Supplement: Datasheet 5: Figure S1 — Maximum likelihood trees of the reductive dehalogenase primer groups for which newly sequenced RdhA were identified, including the new RdhAs and the reductive dehalogenase curated dataset sequences. Sequences for each primer group or set of groups were aligned using Muscle v. 3.8.31 (Edgar, 2004), and the alignments manually curated. Trees were generated in PhyML (Guindon and Gascuel, 2003) under the LG + γ model of amino acid evolution. For each alignment, an outgroup RdhA sequence was included to root the trees (not shown). Names for newly sequenced RdhA genes correspond to the contig from the idba_ud assemblies (Peng et al., 2012), with accession numbers in parentheses. [file DataSheet5.PDF]

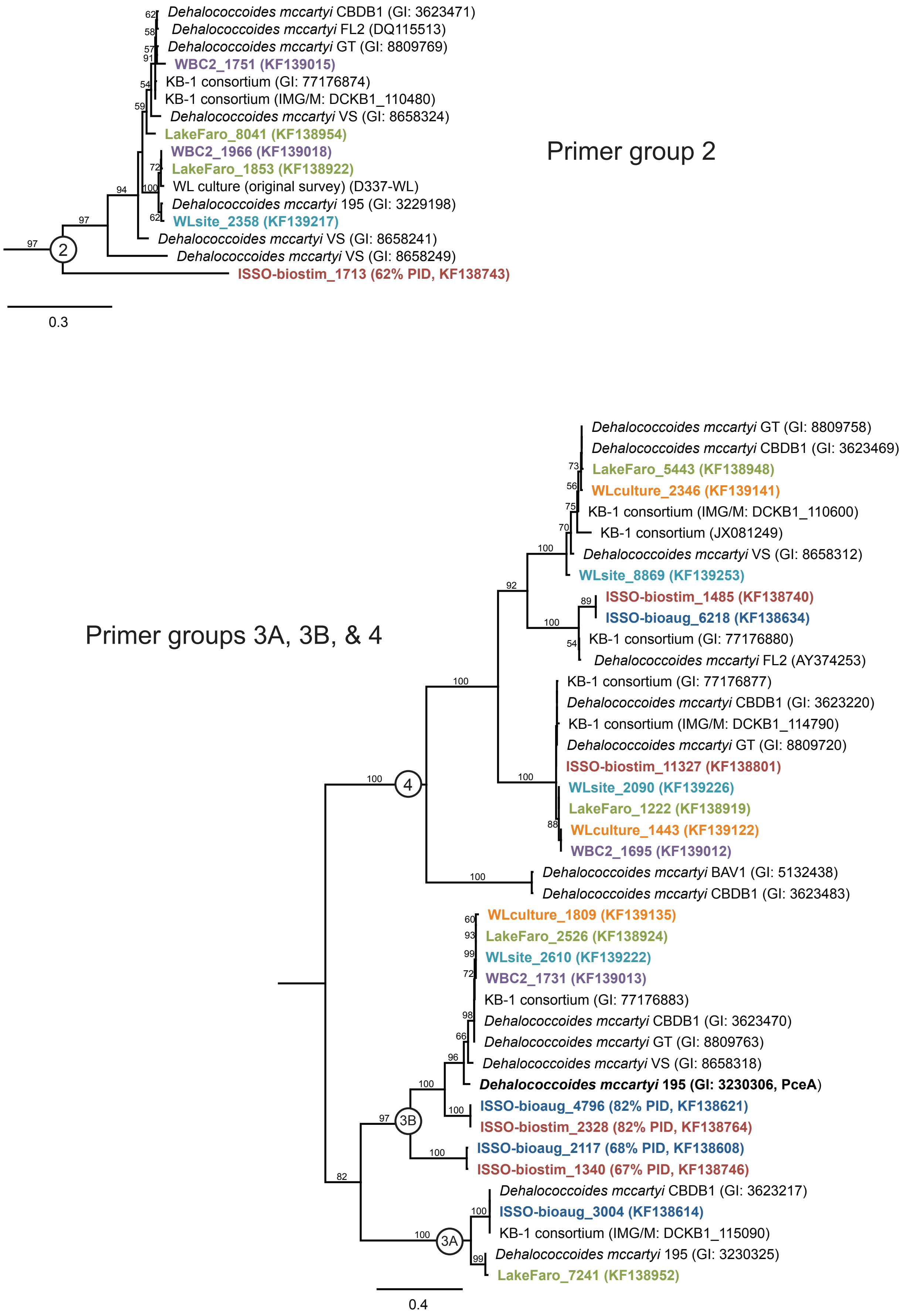

Primer group 2
Dehalococcoides mccartyi CBDB1 (GI: 3623471)
Dehalococcoides mccartyi FL2 (DQ115513)
Dehalococcoides mccartyi GT (GI: 8809769)
WBC2_1751 (KF139015)
KB-1 consortium (GI: 77176874)
KB-1 consortium (IMG/M: DCKB1_110480)
Dehalococcoides mccartyi VS (GI: 8658324)
LakeFaro_8041 (KF138954)
WBC2_1966 (KF139018)
LakeFaro_1853 (KF138922)
WL culture (original survey) (D337-WL)
Dehalococcoides mccartyi 195 (GI: 3229198)
WLsite_2358 (KF139217)
Dehalococcoides mccartyi VS (GI: 8658241)
Dehalococcoides mccartyi VS (GI: 8658249)
ISSO-biostim_1713 (62% PID, KF138743)
0.3
Primer groups 3A, 3B, & 4
Dehalococcoides mccartyi GT (GI: 8809758)
Dehalococcoides mccartyi CBDB1 (GI: 3623469)
LakeFaro_5443 (KF138948)
WLculture_2346 (KF139141)
KB-1 consortium (IMG/M: DCKB1_110600)
KB-1 consortium (JX081249)
Dehalococcoides mccartyi VS (GI: 8658312)
WLsite_8869 (KF139253)
ISSO-biostim_1485 (KF138740)
ISSO-bioaug_6218 (KF138634)
KB-1 consortium (GI: 77176880)
Dehalococcoides mccartyi FL2 (AY374253)
KB-1 consortium (GI: 77176877)
Dehalococcoides mccartyi CBDB1 (GI: 3623220)
KB-1 consortium (IMG/M: DCKB1_114790)
Dehalococcoides mccartyi GT (GI: 8809720)
ISSO-biostim_11327 (KF138801)
WLsite_2090 (KF139226)
LakeFaro_1222 (KF138919)
WLculture_1443 (KF139122)
WBC2_1695 (KF139012)
Dehalococcoides mccartyi BAV1 (GI: 5132438)
Dehalococcoides mccartyi CBDB1 (GI: 3623483)
WLculture_1809 (KF139135)
LakeFaro_2526 (KF138924)
WLsite_2610 (KF139222)
WBC2_1731 (KF139013)
KB-1 consortium (GI: 77176883)
Dehalococcoides mccartyi CBDB1 (GI: 3623470)
Dehalococcoides mccartyi GT (GI: 8809763)
Dehalococcoides mccartyi VS (GI: 8658318)
Dehalococcoides mccartyi 195 (GI: 3230306, PceA)
ISSO-bioaug_4796 (82% PID, KF138621)
ISSO-biostim_2328 (82% PID, KF138764)
ISSO-bioaug_2117 (68% PID, KF138608)
ISSO-biostim_1340 (67% PID, KF138746)
Dehalococcoides mccartyi CBDB1 (GI: 3623217)
ISSO-bioaug_3004 (KF138614)
KB-1 consortium (IMG/M: DCKB1_115090)
Dehalococcoides mccartyi 195 (GI: 3230325)
LakeFaro_7241 (KF138952)
0.4

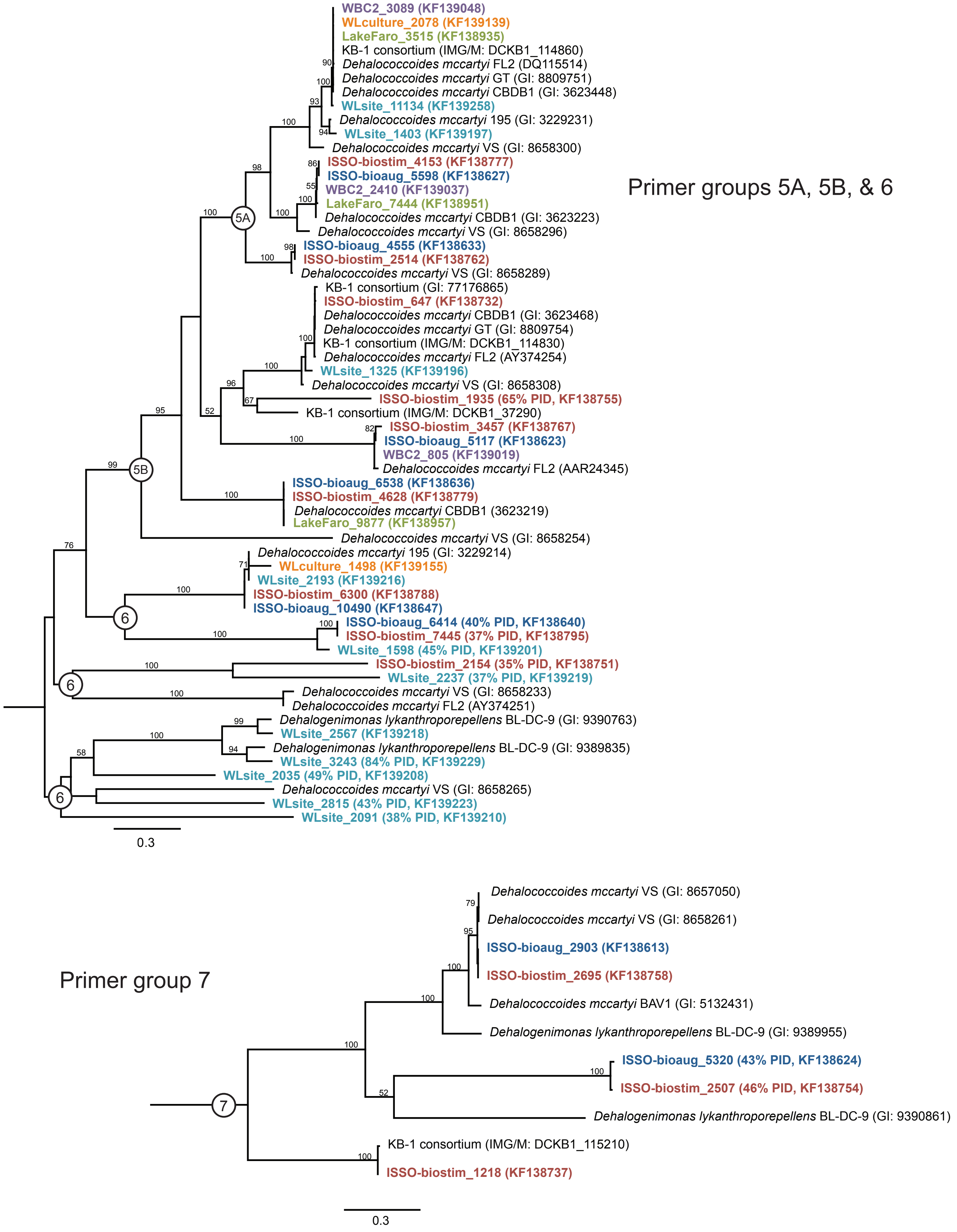

Primer groups 5A, 5B, & 6
WBC2_3089 (KF139048)
WLculture_2078 (KF139139)
LakeFaro_3515 (KF138935)
KB-1 consortium (IMG/M: DCKB1_114860)
Dehalococcoides mccartyi FL2 (DQ115514)
Dehalococcoides mccartyi GT (GI: 8809751)
Dehalococcoides mccartyi CBDB1 (GI: 3623448)
WLsite_11134 (KF139258)
Dehalococcoides mccartyi 195 (GI: 3229231)
WLsite_1403 (KF139197)
Dehalococcoides mccartyi VS (GI: 8658300)
ISSO-biostim_4153 (KF138777)
ISSO-bioaug_5598 (KF138627)
WBC2_2410 (KF139037)
LakeFaro_7444 (KF138951)
Dehalococcoides mccartyi CBDB1 (GI: 3623223)
Dehalococcoides mccartyi VS (GI: 8658296)
ISSO-bioaug_4555 (KF138633)
ISSO-biostim_2514 (KF138762)
Dehalococcoides mccartyi VS (GI: 8658289)
KB-1 consortium (GI: 77176865)
ISSO-biostim_647 (KF138732)
Dehalococcoides mccartyi CBDB1 (GI: 3623468)
Dehalococcoides mccartyi GT (GI: 8809754)
KB-1 consortium (IMG/M: DCKB1_114830)
Dehalococcoides mccartyi FL2 (AY374254)
WLsite_1325 (KF139196)
Dehalococcoides mccartyi VS (GI: 8658308)
ISSO-biostim_1935 (65% PID, KF138755)
KB-1 consortium (IMG/M: DCKB1_37290)
ISSO-biostim_3457 (KF138767)
ISSO-bioaug_5117 (KF138623)
WBC2_805 (KF139019)
Dehalococcoides mccartyi FL2 (AAR24345)
ISSO-bioaug_6538 (KF138636)
ISSO-biostim_4628 (KF138779)
Dehalococcoides mccartyi CBDB1 (3623219)
LakeFaro_9877 (KF138957)
Dehalococcoides mccartyi VS (GI: 8658254)
Dehalococcoides mccartyi 195 (GI: 3229214)
WLculture_1498 (KF139155)
WLsite_2193 (KF139216)
ISSO-biostim_6300 (KF138788)
ISSO-bioaug_10490 (KF138647)
ISSO-bioaug_6414 (40% PID, KF138640)
ISSO-biostim_7445 (37% PID, KF138795)
WLsite_1598 (45% PID, KF139201)
ISSO-biostim_2154 (35% PID, KF138751)
WLsite_2237 (37% PID, KF139219)
Dehalococcoides mccartyi VS (GI: 8658233)
Dehalococcoides mccartyi FL2 (AY374251)
Dehalogenimonas lykanthroporepellens BL-DC-9 (GI: 9390763)
WLsite_2567 (KF139218)
Dehalogenimonas lykanthroporepellens BL-DC-9 (GI: 9389835)
WLsite_3243 (84% PID, KF139229)
WLsite_2035 (49% PID, KF139208)
Dehalococcoides mccartyi VS (GI: 8658265)
WLsite_2815 (43% PID, KF139223)
WLsite_2091 (38% PID, KF139210)
5A
5B
6
0.3
Primer group 7
Dehalococcoides mccartyi VS (GI: 8657050)
Dehalococcoides mccartyi VS (GI: 8658261)
ISSO-bioaug_2903 (KF138613)
ISSO-biostim_2695 (KF138758)
Dehalococcoides mccartyi BAV1 (GI: 5132431)
Dehalogenimonas lykanthroporepellens BL-DC-9 (GI: 9389955)
ISSO-bioaug_5320 (43% PID, KF138624)
ISSO-biostim_2507 (46% PID, KF138754)
Dehalogenimonas lykanthroporepellens BL-DC-9 (GI: 9390861)
KB-1 consortium (IMG/M: DCKB1_115210)
ISSO-biostim_1218 (KF138737)
7
0.3

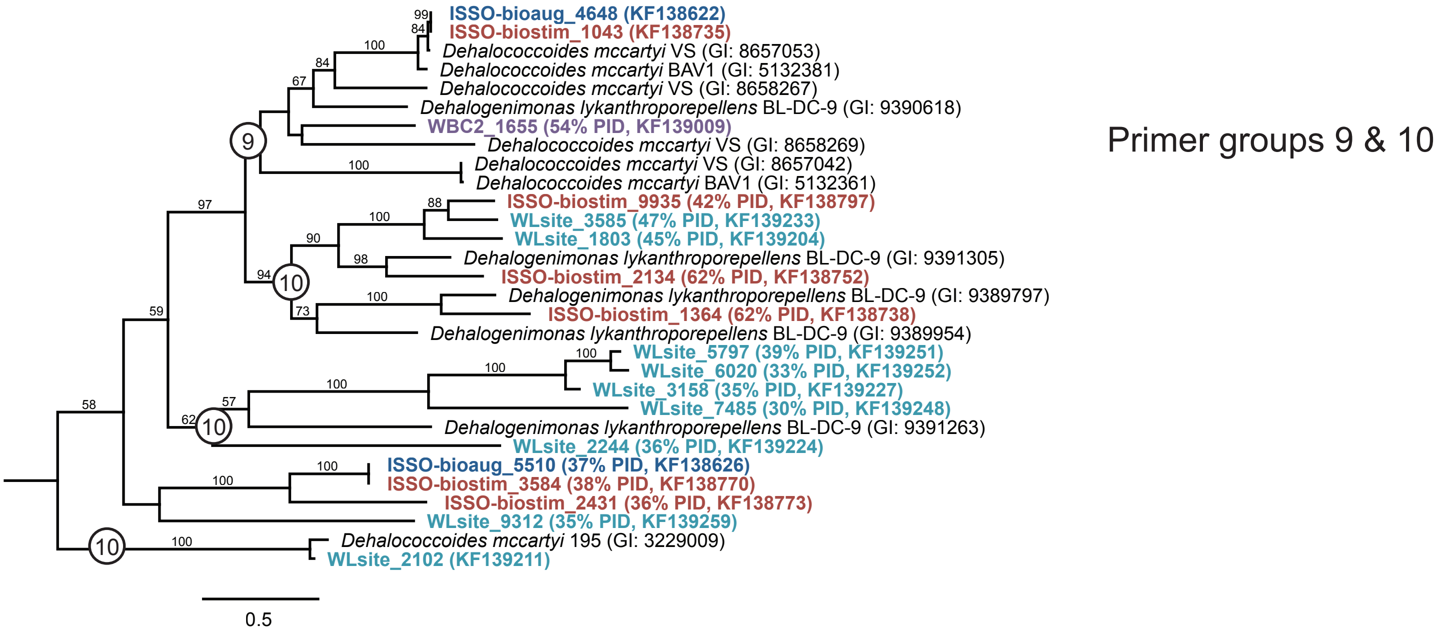
Primer groups 9 & 10
ISSO-bioaug_4648 (KF138622)
ISSO-biostim_1043 (KF138735)
Dehalococcoides mccartyi VS (GI: 8657053)
Dehalococcoides mccartyi BAV1 (GI: 5132381)
Dehalococcoides mccartyi VS (GI: 8658267)
Dehalogenimonas lykanthroporepellens BL-DC-9 (GI: 9390618)
WBC2_1655 (54% PID, KF139009)
Dehalococcoides mccartyi VS (GI: 8658269)
Dehalococcoides mccartyi VS (GI: 8657042)
Dehalococcoides mccartyi BAV1 (GI: 5132361)
ISSO-biostim_9935 (42% PID, KF138797)
WLsite_3585 (47% PID, KF139233)
WLsite_1803 (45% PID, KF139204)
Dehalogenimonas lykanthroporepellens BL-DC-9 (GI: 9391305)
ISSO-biostim_2134 (62% PID, KF138752)
Dehalogenimonas lykanthroporepellens BL-DC-9 (GI: 9389797)
ISSO-biostim_1364 (62% PID, KF138738)
Dehalogenimonas lykanthroporepellens BL-DC-9 (GI: 9389954)
WLsite_5797 (39% PID, KF139251)
WLsite_6020 (33% PID, KF139252)
WLsite_3158 (35% PID, KF139227)
WLsite_7485 (30% PID, KF139248)
Dehalogenimonas lykanthroporepellens BL-DC-9 (GI: 9391263)
WLsite_2244 (36% PID, KF139224)
ISSO-bioaug_5510 (37% PID, KF138626)
ISSO-biostim_3584 (38% PID, KF138770)
ISSO-biostim_2431 (36% PID, KF138773)
WLsite_9312 (35% PID, KF139259)
Dehalococcoides mccartyi 195 (GI: 3229009)
WLsite_2102 (KF139211)
0.5

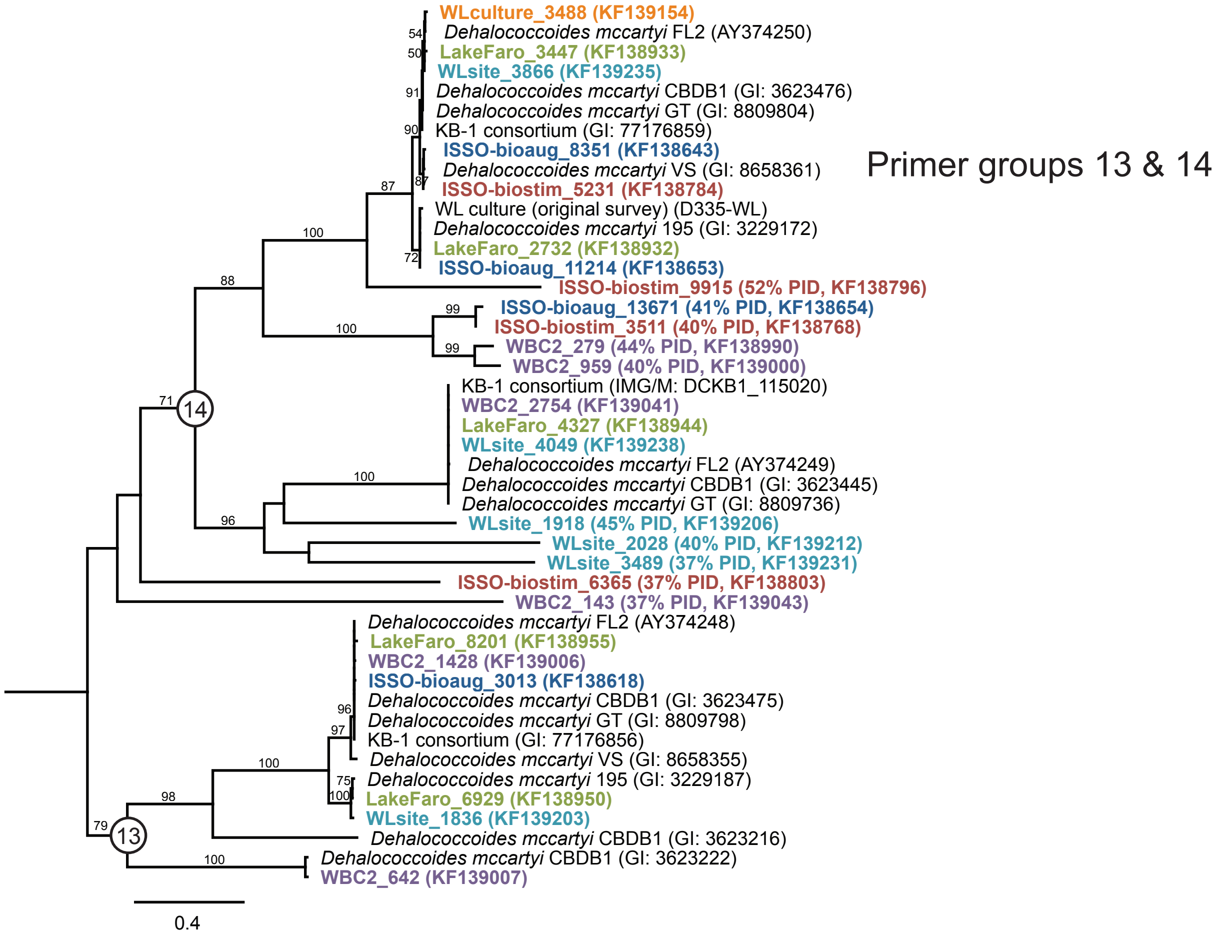
Primer groups 13 & 14
WLculture_3488 (KF139154)
Dehalococcoides mccartyi FL2 (AY374250)
LakeFaro_3447 (KF138933)
WLsite_3866 (KF139235)
Dehalococcoides mccartyi CBDB1 (GI: 3623476)
Dehalococcoides mccartyi GT (GI: 8809804)
KB-1 consortium (GI: 77176859)
ISSO-bioaug_8351 (KF138643)
Dehalococcoides mccartyi VS (GI: 8658361)
ISSO-biostim_5231 (KF138784)
WL culture (original survey) (D335-WL)
Dehalococcoides mccartyi 195 (GI: 3229172)
LakeFaro_2732 (KF138932)
ISSO-bioaug_11214 (KF138653)
ISSO-biostim_9915 (52% PID, KF138796)
ISSO-bioaug_13671 (41% PID, KF138654)
ISSO-biostim_3511 (40% PID, KF138768)
WBC2_279 (44% PID, KF138990)
WBC2_959 (40% PID, KF139000)
KB-1 consortium (IMG/M: DCKB1_115020)
WBC2_2754 (KF139041)
LakeFaro_4327 (KF138944)
WLsite_4049 (KF139238)
Dehalococcoides mccartyi FL2 (AY374249)
Dehalococcoides mccartyi CBDB1 (GI: 3623445)
Dehalococcoides mccartyi GT (GI: 8809736)
WLsite_1918 (45% PID, KF139206)
WLsite_2028 (40% PID, KF139212)
WLsite_3489 (37% PID, KF139231)
ISSO-biostim_6365 (37% PID, KF138803)
WBC2_143 (37% PID, KF139043)
Dehalococcoides mccartyi FL2 (AY374248)
LakeFaro_8201 (KF138955)
WBC2_1428 (KF139006)
ISSO-bioaug_3013 (KF138618)
Dehalococcoides mccartyi CBDB1 (GI: 3623475)
Dehalococcoides mccartyi GT (GI: 8809798)
KB-1 consortium (GI: 77176856)
Dehalococcoides mccartyi VS (GI: 8658355)
Dehalococcoides mccartyi 195 (GI: 3229187)
LakeFaro_6929 (KF138950)
WLsite_1836 (KF139203)
Dehalococcoides mccartyi CBDB1 (GI: 3623216)
Dehalococcoides mccartyi CBDB1 (GI: 3623222)
WBC2_642 (KF139007)
0.4

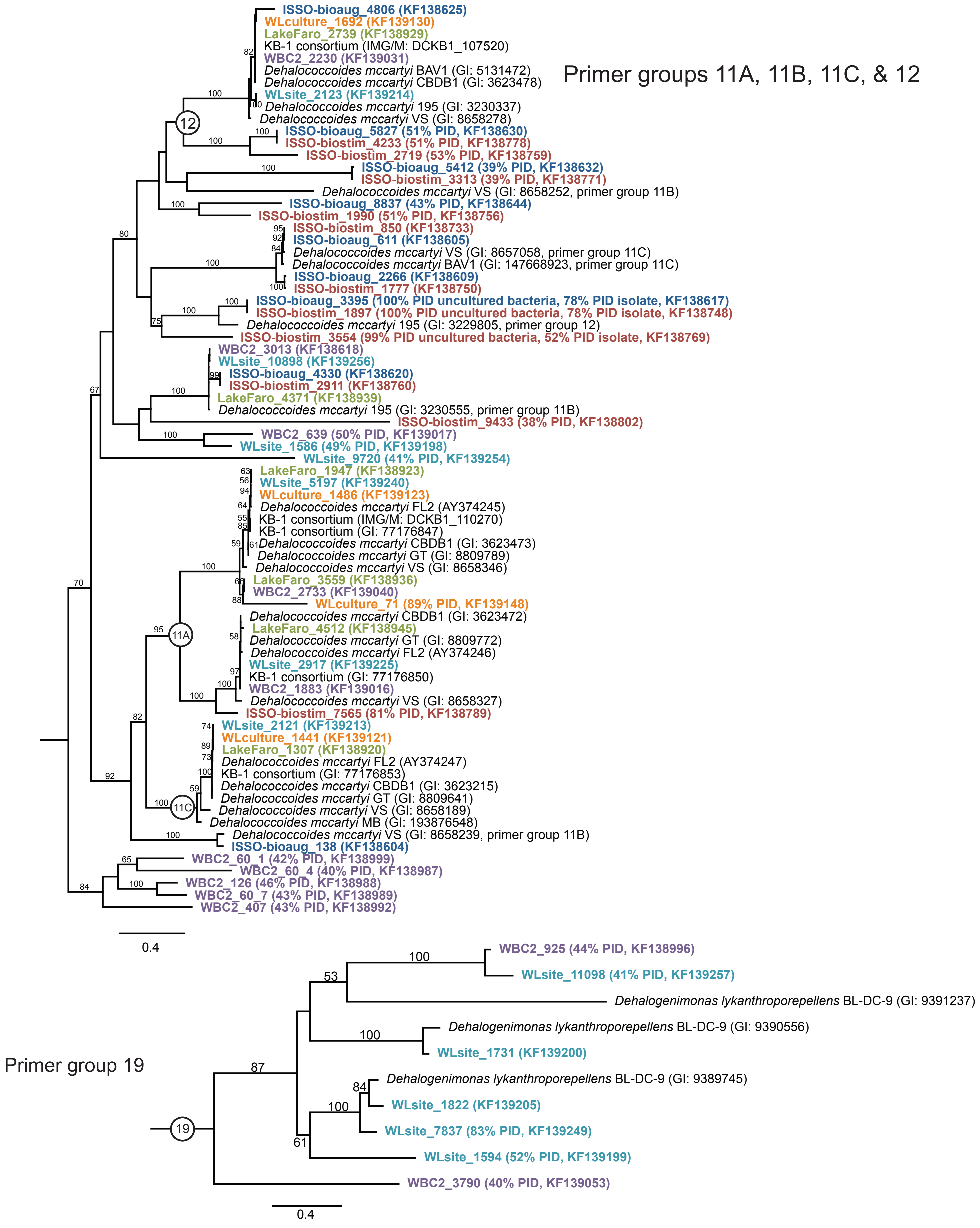

Primer groups 11A, 11B, 11C, & 12
ISSO-bioaug_4806 (KF138625)
WLculture_1692 (KF139130)
LakeFaro_2739 (KF138929)
KB-1 consortium (IMG/M: DCKB1_107520)
WBC2_2230 (KF139031)
Dehalococcoides mccartyi BAV1 (GI: 5131472)
Dehalococcoides mccartyi CBDB1 (GI: 3623478)
WLsite_2123 (KF139214)
Dehalococcoides mccartyi 195 (GI: 3230337)
Dehalococcoides mccartyi VS (GI: 8658278)
12
ISSO-bioaug_5827 (51% PID, KF138630)
ISSO-biostim_4233 (51% PID, KF138778)
ISSO-biostim_2719 (53% PID, KF138759)
ISSO-bioaug_5412 (39% PID, KF138632)
ISSO-biostim_3313 (39% PID, KF138771)
Dehalococcoides mccartyi VS (GI: 8658252, primer group 11B)
ISSO-bioaug_8837 (43% PID, KF138644)
ISSO-biostim_1990 (51% PID, KF138756)
ISSO-biostim_850 (KF138733)
ISSO-bioaug_611 (KF138605)
Dehalococcoides mccartyi VS (GI: 8657058, primer group 11C)
Dehalococcoides mccartyi BAV1 (GI: 147668923, primer group 11C)
ISSO-bioaug_2266 (KF138609)
ISSO-biostim_1777 (KF138750)
ISSO-bioaug_3395 (100% PID uncultured bacteria, 78% PID isolate, KF138617)
ISSO-biostim_1897 (100% PID uncultured bacteria, 78% PID isolate, KF138748)
Dehalococcoides mccartyi 195 (GI: 3229805, primer group 12)
ISSO-biostim_3554 (99% PID uncultured bacteria, 52% PID isolate, KF138769)
WBC2_3013 (KF138618)
WLsite_10898 (KF139256)
ISSO-bioaug_4330 (KF138620)
ISSO-biostim_2911 (KF138760)
LakeFaro_4371 (KF138939)
Dehalococcoides mccartyi 195 (GI: 3230555, primer group 11B)
ISSO-biostim_9433 (38% PID, KF138802)
WBC2_639 (50% PID, KF139017)
WLsite_1586 (49% PID, KF139198)
WLsite_9720 (41% PID, KF139254)
LakeFaro_1947 (KF138923)
WLsite_5197 (KF139240)
WLculture_1486 (KF139123)
Dehalococcoides mccartyi FL2 (AY374245)
KB-1 consortium (IMG/M: DCKB1_110270)
KB-1 consortium (GI: 77176847)
Dehalococcoides mccartyi CBDB1 (GI: 3623473)
Dehalococcoides mccartyi GT (GI: 8809789)
Dehalococcoides mccartyi VS (GI: 8658346)
LakeFaro_3559 (KF138936)
WBC2_2733 (KF139040)
WLculture_71 (89% PID, KF139148)
11A
Dehalococcoides mccartyi CBDB1 (GI: 3623472)
LakeFaro_4512 (KF138945)
Dehalococcoides mccartyi GT (GI: 8809772)
Dehalococcoides mccartyi FL2 (AY374246)
WLsite_2917 (KF139225)
KB-1 consortium (GI: 77176850)
WBC2_1883 (KF139016)
Dehalococcoides mccartyi VS (GI: 8658327)
ISSO-biostim_7565 (81% PID, KF138789)
WLsite_2121 (KF139213)
WLculture_1441 (KF139121)
LakeFaro_1307 (KF138920)
Dehalococcoides mccartyi FL2 (AY374247)
KB-1 consortium (GI: 77176853)
Dehalococcoides mccartyi CBDB1 (GI: 3623215)
Dehalococcoides mccartyi GT (GI: 8809641)
11C
Dehalococcoides mccartyi VS (GI: 8658189)
Dehalococcoides mccartyi MB (GI: 193876548)
Dehalococcoides mccartyi VS (GI: 8658239, primer group 11B)
ISSO-bioaug_138 (KF138604)
WBC2_60_1 (42% PID, KF138999)
WBC2_60_4 (40% PID, KF138987)
WBC2_126 (46% PID, KF138988)
WBC2_60_7 (43% PID, KF138989)
WBC2_407 (43% PID, KF138992)
0.4
Primer group 19
WBC2_925 (44% PID, KF138996)
WLsite_11098 (41% PID, KF139257)
Dehalogenimonas lykanthroporepellens BL-DC-9 (GI: 9391237)
Dehalogenimonas lykanthroporepellens BL-DC-9 (GI: 9390556)
WLsite_1731 (KF139200)
19
Dehalogenimonas lykanthroporepellens BL-DC-9 (GI: 9389745)
WLsite_1822 (KF139205)
WLsite_7837 (83% PID, KF139249)
WLsite_1594 (52% PID, KF139199)
WBC2_3790 (40% PID, KF139053)
0.4

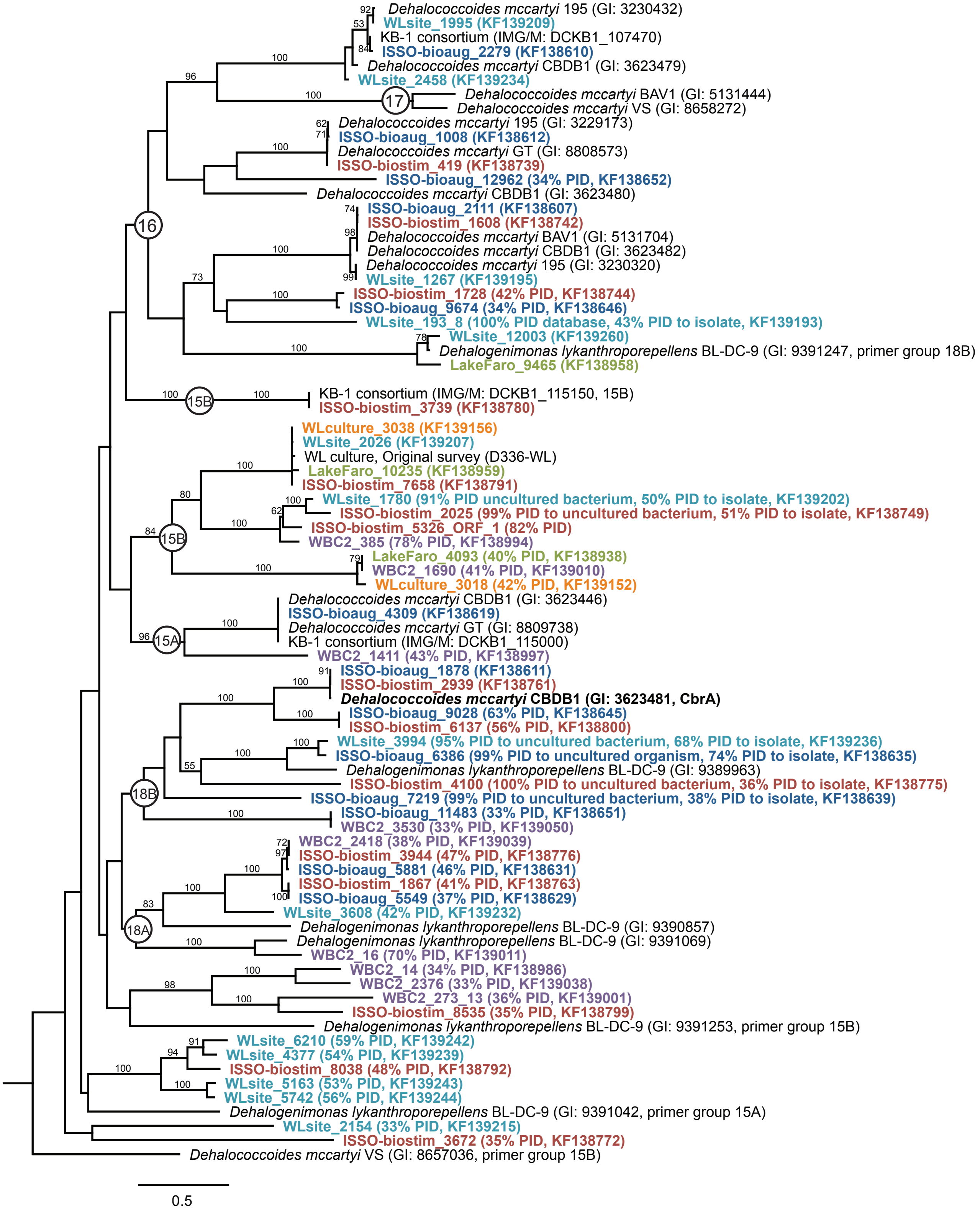

Primer groups 15A, 15B, 16, 17, 18A, & 18B

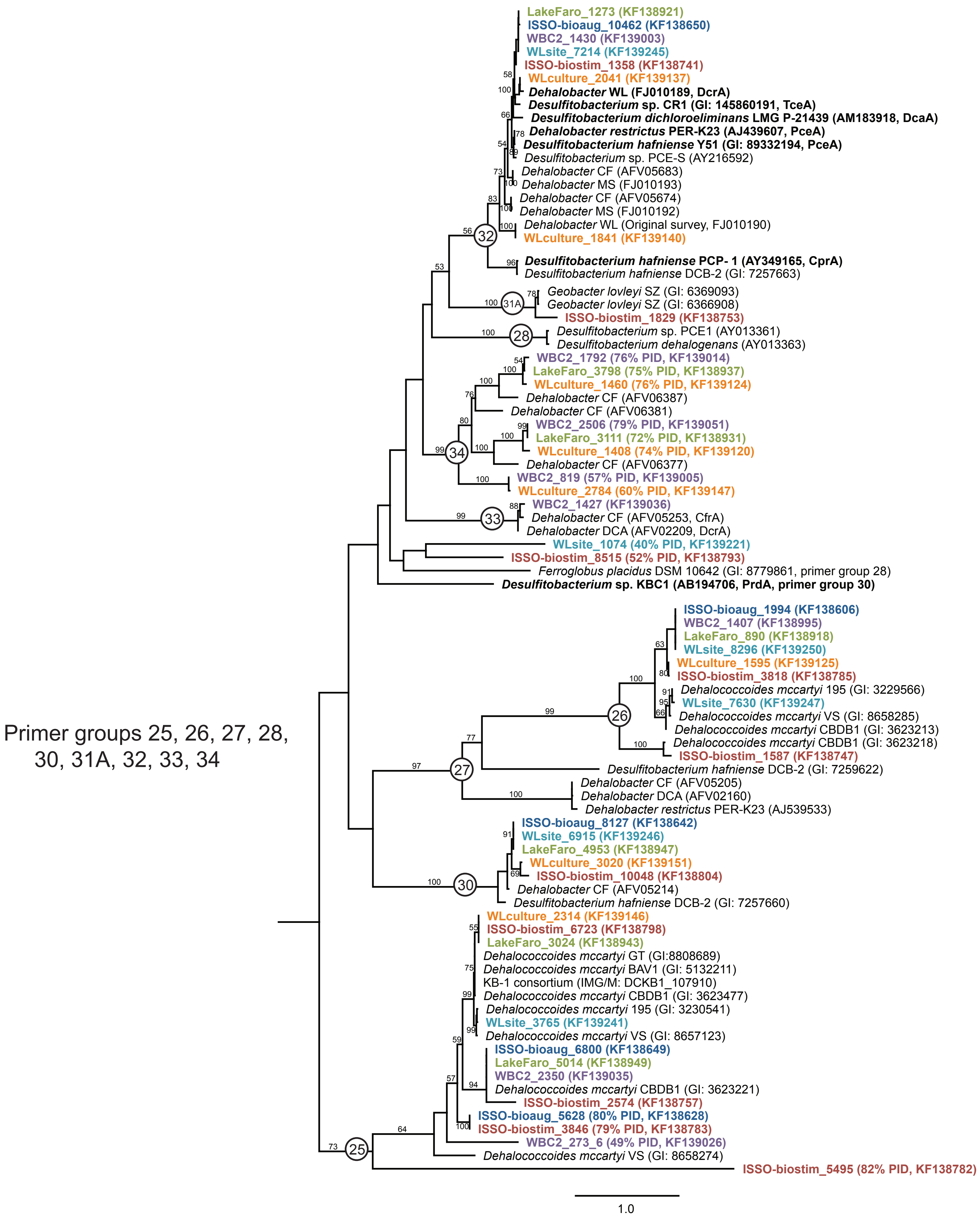
LakeFaro_1273 (KF138921)
ISSO-bioaug_10462 (KF138650)
WBC2_1430 (KF139003)
WLsite_7214 (KF139245)
ISSO-biostim_1358 (KF138741)
WLculture_2041 (KF139137)
Dehalobacter WL (FJ010189, DcrA)
Desulfitobacterium sp. CR1 (GI: 145860191, TceA)
Desulfitobacterium dichloroeliminans LMG P-21439 (AM183918, DcaA)
Dehalobacter restrictus PER-K23 (AJ439607, PceA)
Desulfitobacterium hafniense Y51 (GI: 89332194, PceA)
Desulfitobacterium sp. PCE-S (AY216592)
Dehalobacter CF (AFV05683)
Dehalobacter MS (FJ010193)
Dehalobacter CF (AFV05674)
Dehalobacter MS (FJ010192)
Dehalobacter WL (Original survey, FJ010190)
WLculture_1841 (KF139140)
32
Desulfitobacterium hafniense PCP- 1 (AY349165, CprA)
Desulfitobacterium hafniense DCB-2 (GI: 7257663)
31A
Geobacter lovleyi SZ (GI: 6369093)
Geobacter lovleyi SZ (GI: 6366908)
ISSO-biostim_1829 (KF138753)
28
Desulfitobacterium sp. PCE1 (AY013361)
Desulfitobacterium dehalogenans (AY013363)
WBC2_1792 (76% PID, KF139014)
LakeFaro_3798 (75% PID, KF138937)
WLculture_1460 (76% PID, KF139124)
Dehalobacter CF (AFV06387)
Dehalobacter CF (AFV06381)
WBC2_2506 (79% PID, KF139051)
LakeFaro_3111 (72% PID, KF138931)
WLculture_1408 (74% PID, KF139120)
34
Dehalobacter CF (AFV06377)
WBC2_819 (57% PID, KF139005)
WLculture_2784 (60% PID, KF139147)
WBC2_1427 (KF139036)
33
Dehalobacter CF (AFV05253, CfrA)
Dehalobacter DCA (AFV02209, DcrA)
WLsite_1074 (40% PID, KF139221)
ISSO-biostim_8515 (52% PID, KF138793)
Ferroglobus placidus DSM 10642 (GI: 8779861, primer group 28)
Desulfitobacterium sp. KBC1 (AB194706, PrdA, primer group 30)
ISSO-bioaug_1994 (KF138606)
WBC2_1407 (KF138995)
LakeFaro_890 (KF138918)
WLsite_8296 (KF139250)
WLculture_1595 (KF139125)
ISSO-biostim_3818 (KF138785)
Dehalococcoides mccartyi 195 (GI: 3229566)
WLsite_7630 (KF139247)
26
Dehalococcoides mccartyi VS (GI: 8658285)
Dehalococcoides mccartyi CBDB1 (GI: 3623213)
Dehalococcoides mccartyi CBDB1 (GI: 3623218)
ISSO-biostim_1587 (KF138747)
Primer groups 25, 26, 27, 28,
30, 31A, 32, 33, 34
27
Desulfitobacterium hafniense DCB-2 (GI: 7259622)
Dehalobacter CF (AFV05205)
Dehalobacter DCA (AFV02160)
Dehalobacter restrictus PER-K23 (AJ539533)
ISSO-bioaug_8127 (KF138642)
WLsite_6915 (KF139246)
LakeFaro_4953 (KF138947)
WLculture_3020 (KF139151)
ISSO-biostim_10048 (KF138804)
30
Dehalobacter CF (AFV05214)
Desulfitobacterium hafniense DCB-2 (GI: 7257660)
WLculture_2314 (KF139146)
ISSO-biostim_6723 (KF138798)
LakeFaro_3024 (KF138943)
Dehalococcoides mccartyi GT (GI:8808689)
Dehalococcoides mccartyi BAV1 (GI: 5132211)
KB-1 consortium (IMG/M: DCKB1_107910)
Dehalococcoides mccartyi CBDB1 (GI: 3623477)
Dehalococcoides mccartyi 195 (GI: 3230541)
WLsite_3765 (KF139241)
Dehalococcoides mccartyi VS (GI: 8657123)
ISSO-bioaug_6800 (KF138649)
LakeFaro_5014 (KF138949)
WBC2_2350 (KF139035)
Dehalococcoides mccartyi CBDB1 (GI: 3623221)
ISSO-biostim_2574 (KF138757)
ISSO-bioaug_5628 (80% PID, KF138628)
ISSO-biostim_3846 (79% PID, KF138783)
WBC2_273_6 (49% PID, KF139026)
25
Dehalococcoides mccartyi VS (GI: 8658274)
ISSO-biostim_5495 (82% PID, KF138782)
1.0
